# Variation in Membrane Trafficking Linked to SNARE AtSYP51 Interaction With Aquaporin NIP1;1

**DOI:** 10.3389/fpls.2018.01949

**Published:** 2019-01-09

**Authors:** Fabrizio Barozzi, Paride Papadia, Giovanni Stefano, Luciana Renna, Federica Brandizzi, Danilo Migoni, Francesco Paolo Fanizzi, Gabriella Piro, Gian-Pietro Di Sansebastiano

**Affiliations:** ^1^Laboratory of Botany, DISTEBA (Diartimento di Scienze e Tecnologie Biologiche e Ambientali), University of Salento, Lecce, Italy; ^2^Laboratory of General and Inorganic Chemistry, DISTEBA (Dipartimento di Scienze e Tecnologie Biologiche e Ambientali), University of Salento, Lecce, Italy; ^3^MSU DOE-Plant Biology Lab, Michigan State University, East Lansing, MI, United States

**Keywords:** SNARE, aquaporin, SYP51, NIP1, rBiFC, tonoplast recycling, tonoplast, endoplasmic reticulum

## Abstract

SYP51 and 52 are the two members of the SYP5 Qc-SNARE gene family in *Arabidopsis thaliana*. These two proteins, besides their high level of sequence identity (85%), have shown to have differential functional specificity and possess a different interactome. Here we describe a unique and specific interaction of SYP51 with an ER aquaporin, AtNIP1;1 (also known as NLM1) indicated to be able to transport arsenite [As(III)] and previously localized on PM. In the present work we investigate in detail such localization *in vivo* and characterize the interaction with SYP51. We suggest that this interaction may reveal a new mechanism regulating tonoplast invagination and recycling. We propose this interaction to be part of a regulatory mechanism associated with direct membrane transport from ER to tonoplast and Golgi mediated vesicle trafficking. We also demonstrate that NIP1;1 is important for plant tolerance to arsenite but does not alter its uptake or translocation. To explain such phenomenon the hypothesis that SYP51/NIP1;1 interaction modifies ER and vacuole ability to accumulate arsenite is discussed.

## Introduction

Membrane traffic regulation is essential to compartmentalization and proper functioning of the cell. Soluble N-ethylmaleimide-sensitive factor attachment protein receptor (SNARE) proteins are the main determinants of membrane fusion specificity and are known to act forming coiled-coil interactions between specific sets of partners (Jahn and Scheller, 2006). Each organelle in the endomembrane system contains a specific group of SNAREs so that specific SNARE-complex formation is thought to provide the specificity of membrane fusion (Jahn and Scheller, 2006). The presence of a glutamine (Q) or arginine (R) residue in the SNARE domain allows to structurally classify these proteins into Q and R groups and Q-SNAREs can be further classified into Qa, Qb, and Qc (Fasshauer et al., 1998). A specific SNARE-complex will require the contribution of Qa, Qb, Qc, and R domains.

Several SNAREs are involved in post-Golgi membrane trafficking to the tonoplast including Qa-SNAREs, SYP21 and 22, Qb-SNAREs, VTI11 and 12, Qc-SNAREs, SYP51 and 52. It was shown that both SYP21 and 22 interact with VTI11 and SYP51 at the prevacuolar compartment and/or at the tonoplast, and the VTI12, SYP41/SYP42, and SYP61 SNAREs form a complex at the TGN (Zheng et al., 1999; Sanderfoot et al., 2001; Surpin et al., 2003; Yano et al., 2003; Ebine et al., 2008). The VTI proteins are partially redundant in function but VTI11 appears more associated to clathrin-dependent transport to the lytic vacuole whereas VTI12 to storage proteins vacuolar traffic (Sanmartín et al., 2007).

SYP51 and 52 are the two members of the SYP5 Qc-SNARE gene family in *Arabidopsis thaliana*. These two proteins have shown to have differential affinity for Qb-VTI11 -VTI12 and VAMP727 (Sanderfoot et al., 2001; Sanmartín et al., 2007; Ebine et al., 2008). The functional specificity of AtSYP51 and AtSYP52 emerged more clearly when the effect on two distinct vacuolar markers GFPgl133Chi and AleuGFP was analyzed (De Benedictis et al., 2013; Faraco et al., 2013). SYP51 dominant negative mutant appeared to affect GFPgl133Chi sorting to the central vacuole more than SYP52. This appeared equally important for GFPgl133Chi and AleuGFP (De Benedictis et al., 2013; Faraco et al., 2013). The localization of these SNAREs was on TGN and tonoplast. Accumulated on the tonoplast, SYP51 showed a clear interfering activity since it reduced the vacuolar targeting of the marker RGUSChi (De Benedictis et al., 2013; Di Sansebastiano, 2013).

To further investigate the functional specificity of these two SYP5 we describe here a specific interaction of SYP51 with an ER aquaporin, AtNIP1;1 (also known as NLM1). AtNIP1;1 is an aquaglyceroporin able to transport arsenite [As(III)] (Kamiya et al., 2009) and antimony (Kamiya and Fujiwara, 2009), previously localized on PM (Kamiya et al., 2009). In the present work we revise AtNIP1;1 reported localization *in vivo* and characterize its interaction with SYP51 deriving important insight into functions of both proteins. This interaction could be the cause of the interfering activity of SYP51 (De Benedictis et al., 2013) and of a new mechanism to regulate tonoplast invagination and recycling. We propose this interaction to be a regulatory mechanism to maintain the balance between direct membrane transport from ER to tonoplast and Golgi mediated vacuolar vesicle traffic.

## Results

### AtSYP51 Specifically Interacts *in vivo* With AtNIP1;1/AtNLM1

Interactions Network analysis was performed starting from Arabidopsis SNAREs and searching only interacting candidates among transmembrane proteins belonging to the secretory system. We started to interrogate the databases using the software Cytoscape 3.4.0 (Shannon et al., 2003) and the data present in the BAR database (Geisler-Lee et al., 2007). Interactions from 53 SNAREs were reported in the database. They produced a network with 1,604 nodes and 2,562 interactions.

**Figure 1:**
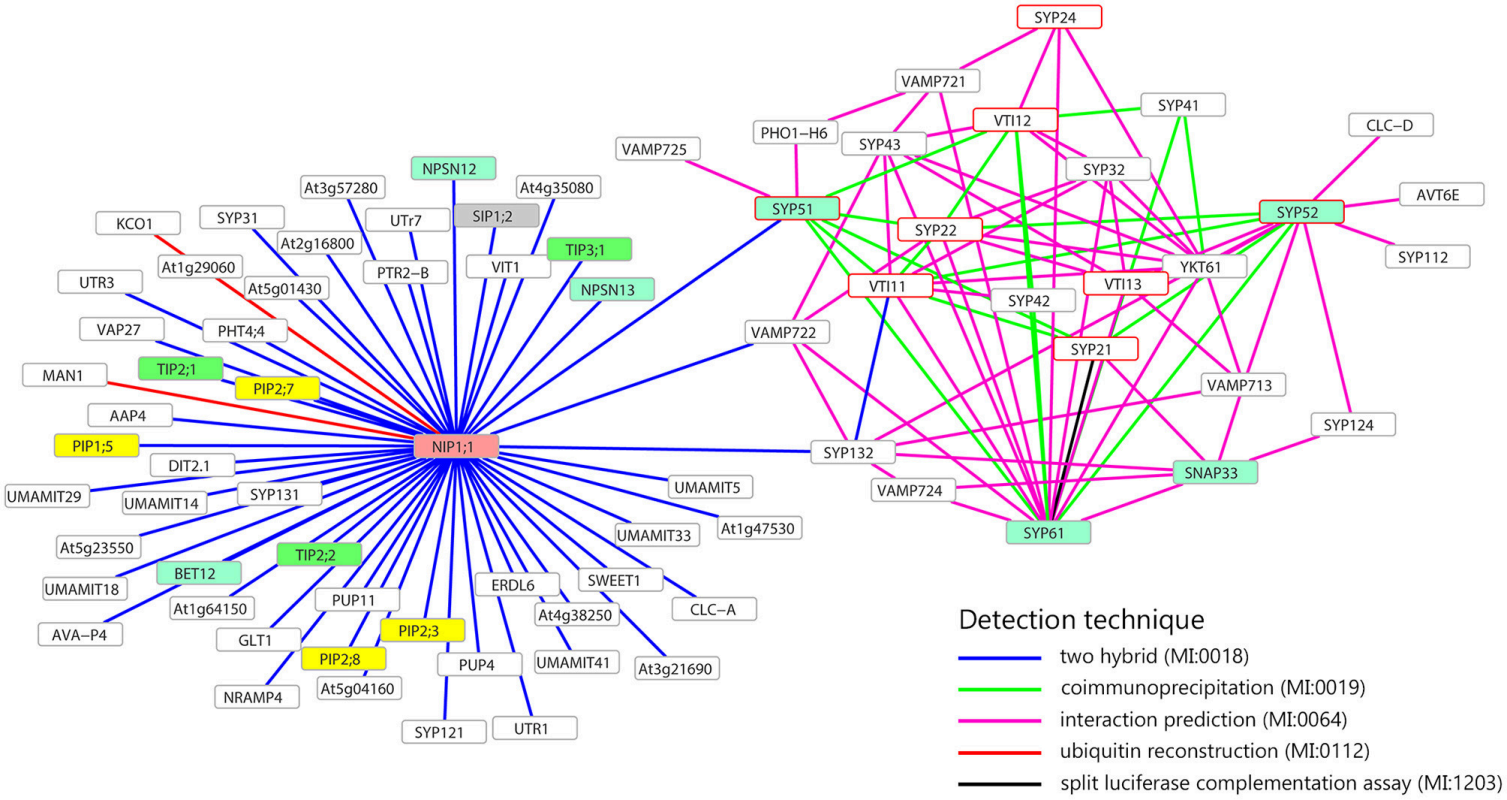
Interaction network of NIP1;1, SYP51, SYP52, and SYP61. Only membrane proteins involved in the transport of small molecules and water (i.e., aquaporin) interacting with NIP1;1, SYP51, SYP52, or SYP61, are shown. Turquoise frames highlight QcSNAREs, red border frames indicates the SNAREs involved in the formation of vacuolar complex. In yellow, green, gray, and pink, respectively PIPs, TIPs, SIPs, NIPs aquaporins. Gray border frames indicate other SNAREs and other kinds of membrane proteins. Data retrieved from BAR database (http://bar.utoronto.ca).

The attention was then focused on SYP5s interactions with some transmembrane proteins (SNAREs, channels and transporters) and the analysis supported the possibility that AtSYP51 interacts with the other SNAREs involved in vacuolar traffic, SYP21, SYP22, SYP61, VTI11, VTI12, and VAMP725 and with the aquaporin NIP1;1 (Figure 1). AtSYP51 was also predicted to interact with the phosphate transporter PHO1-H6 likely expressed in the connective tissues of the stamen (Wang et al., 2004) (Full list of interactors in Supplementary Table 1).

The interaction between SYP51 and NIP1;1 appeared highly specific since the homologous gene *SYP52* which shares 85% identity with *SYP51*, did not interact. SYP52 was predicted to interact with the other partner SNAREs (SYP21, SYP22, SYP61, SYP112, SYP122, SYP132, VTI11, VTI13, VAMP713), YKT61 and other transmembrane proteins with potential effects on traffic: AVT6E, an amino acid transporter predominantly accumulated at the ER but also partially localized to the vacuolar membrane (Fujiki et al., 2017) and CLC-d, an anion's transporter localized in the TGN where it controls luminal pH, mediating the transport of a counter anions' such as Cl^−^ or NO$NO_{3}{}^{-}$ (von der Fecht-Bartenbach et al., 2007).

**Figure 2:**
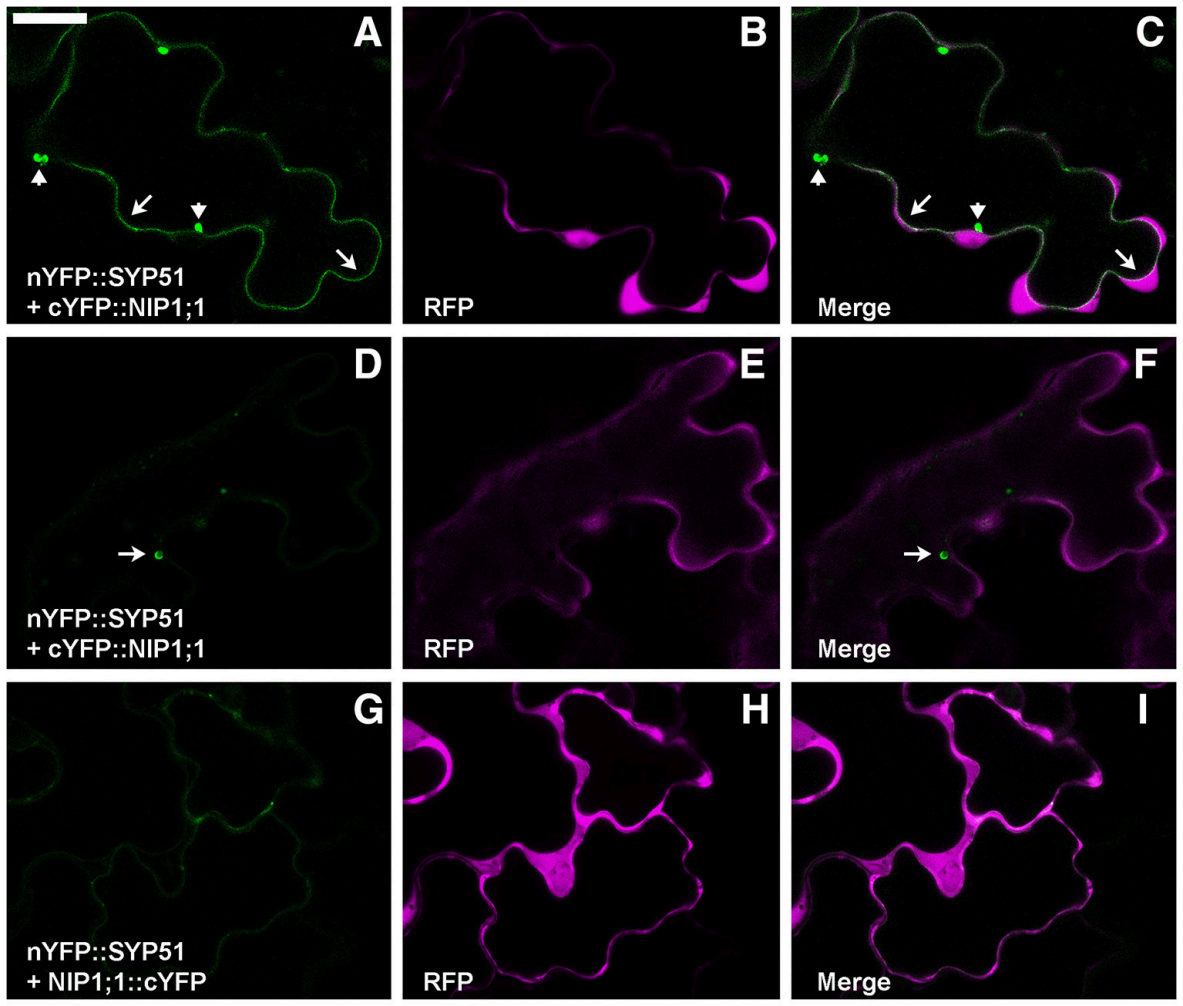
Epidermal cells' confocal images of Arabidopsis cotyledons transiently co-expressing rBiFC constructs. **(A)** nYFP::SYP51 strongly interacts with cYFP::NIP1;1, labeling with YFP fluorescence both tonoplast (arrows) and small aggregates (arrowheads); **(B)** RFP is expressed and dispersed in the cytosol; **(C)** overlay of images **(A,B)**; **(D)** cells expressing nYFP::SYP51 and cYFP::NIP1;1 occasionally show more intense fluorescence in “donut-like structure” (arrow) than in the tonoplast; **(E)** RFP signal is well visible; **(F)** overlay of images **(D,E)**; **(G)** nYFP::SYP51 weakly interacts with NIP1;1::cYFP, producing not well defined, diffused signal; **(H)** RFP signal is always proportionally much higher than YFP. The other constructs produce much less or no YFP fluorescence at all (partially shown in Figure 10). **(I)** Overlay of images **(G,H)**; Scale bar = 20 μm.

To test the putative SYP51/NIP1;1 interaction *in vivo* we used the technique of Ratiometric (using cytosolic RFP) Bimolecular Fluorescence complementation (rBiFC) (Grefen and Blatt, 2012; Xing et al., 2016). A set of controls was added to SYP51, including the highly homolog SYP52, the similar Qc-SNARE SYP61 and the more distant Qa-SNARE SYP122. Split YFP was fused at the N-terminus of SNAREs and the complementary part was fused alternatively to N- and C-terminus of NIP1;1. Only nYFP::SYP51 showed a strong interaction with cYFP::NIP1;1. The reconstituted fluorescent signal labeled the tonoplast and small associated aggregates (Figures 2A–C). In some cells, it was possible to observe more intense interaction in small compartments other than on the tonoplast. We decided to name these “donut-like structures” because they seem similar to those observed in other studies (Jaillais et al., 2006; Honig et al., 2012). The observation, repeated in several occasions, gave no clues if such structures corresponded to aggregates observed also in other cells, but we noticed that dimension ranged only between 2 and 5 μm and did not appear multivesicular (Figures 2D–F). Among the controls, both nYFP::SYP51 (Figures 2G–I) and nYPF-SYP52 weakly interacted with NIP1;1::cYFP but similar chimerical constructs with GFP at the C-terminus of NIP1 were found to be dispersed in the cytosol (Supplementary Figure 1A).

### *In vivo* Localization of XFP::AtNIP1;1

AtNIP1;1 subcellular distribution was studied with chimerical constructs with fluorescent tags (GFP or RFP) and co-expression with known markers. The fusion of the tag to the C-terminus showed to induce mis-localization of the protein in the cytosol preventing the insertion in the membrane (Supplementary Figure 1A). The fusion of GFP to the N-terminus showed to produce a stable and membrane associated construct (Supplementary Figure 1B) and we also verified that RFP::NIP1;1 distribution was identical (Supplementary Figure 1C).

Transgenic plants expressing *RFP::NIP1;1* under the control of the CaMV35S promoter, were produced to verify the protein accumulation (Supplementary Figure 2) in different tissues. The results showed no differences between distribution of fluorescent proteins in stable transformants (Supplementary Figures 3, 4) and transient expression. Co-localization experiments were performed with transient transformation assays in cotyledons using RFP::NIP1;1.

**Figure 3:**
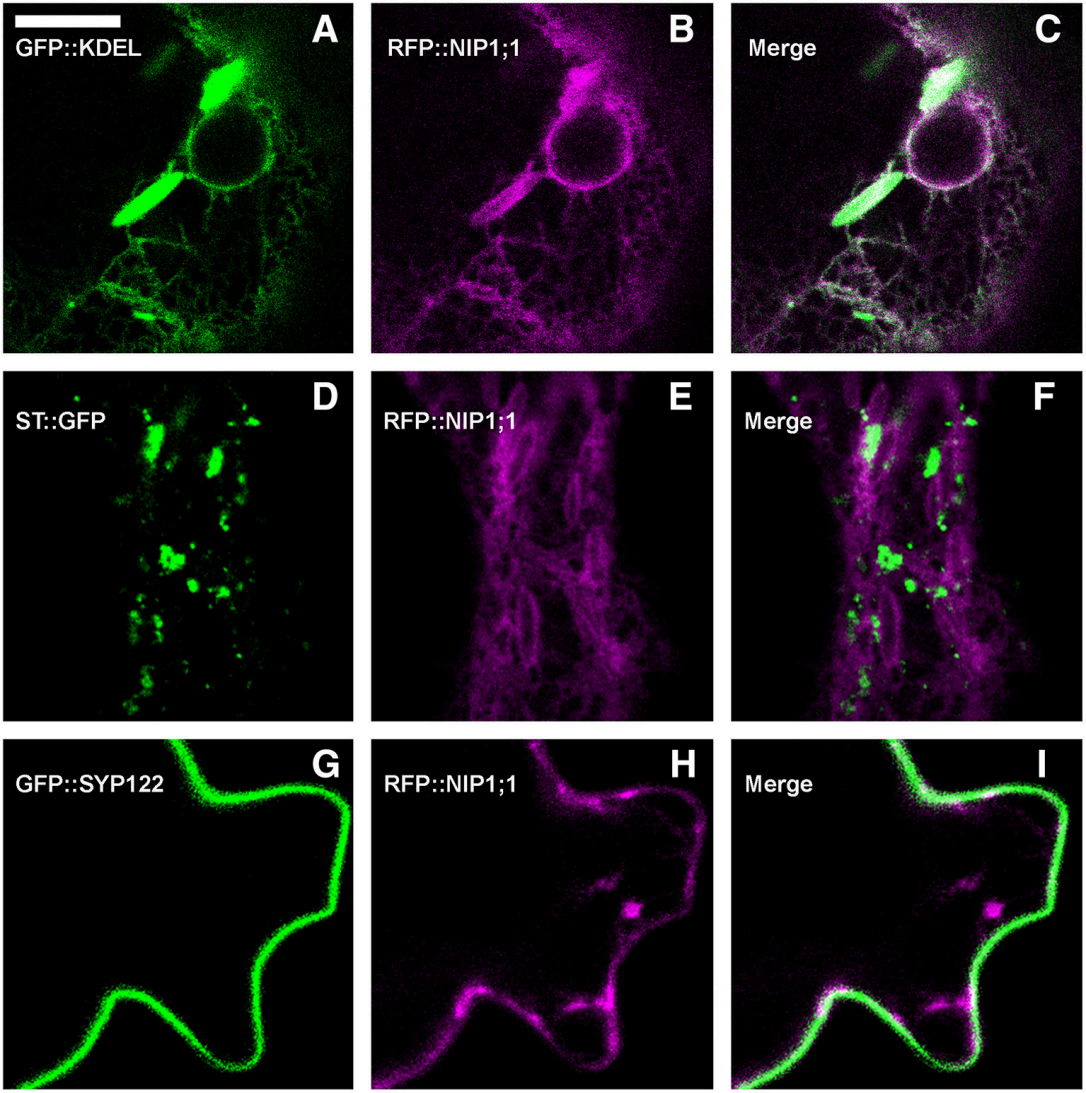
Epidermal cells' confocal images of Arabidopsis cotyledons, transiently co-expressing RFP::NIP1;1 with **(A–C)** ER marker GFP::KDEL; **(D–F)** Golgi marker ST::GFP; **(G–I)** plasma membrane marker GFP::SYP122. The first column shows GFP signal in green, the second column shows RFP signal in magenta, whereas the third column shows the merge of fluorescent signals and co-localization in white. Scale bar = 10 μm.

**Figure 4:**
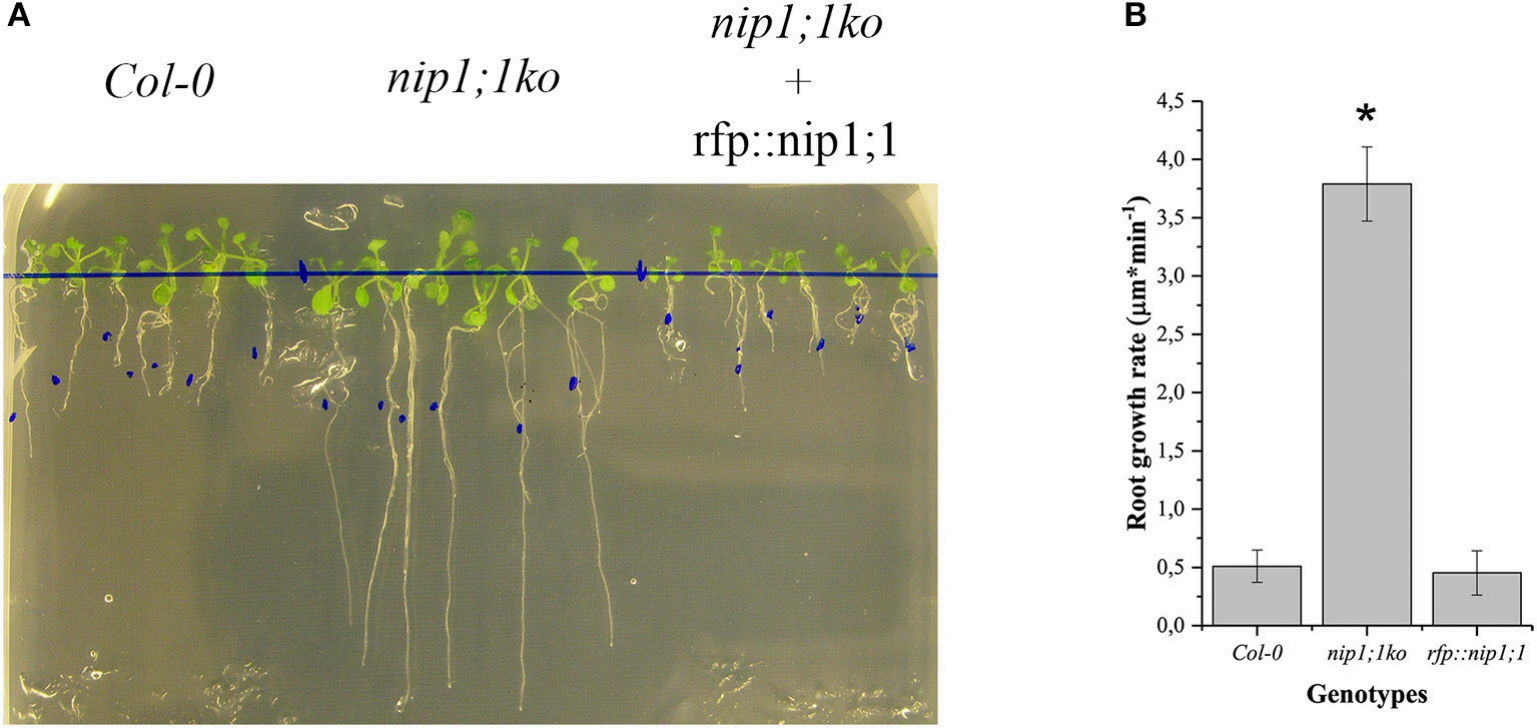
Comparison of Arabidopsis Col0 wild-type nip1;1ko and nip1;1ko complemented by the expression of RFP::NIP1;1 (rfp::nip1;1) grown on MS medium with 15 μM As(III). **(A)** Plants growing on the same medium; the blue dots mark the length of the root at the start of experiment. **(B)** Root growth speed average (*n* = 5), significance (*) expressed as *p* = 8.29·10^−10^. 5 replicates correspond to 50 plants.

RFP::NIP1;1 distribution overlapped with GFP::KDEL at the ER (Figures 3A–C) but it labeled the membrane while GFP::KDEL was soluble in the lumen of ER-bodies (Matsushima et al., 2003). RFP::NIP1;1 did not co-localize either with the Golgi marker ST::GFP (Figures 3D–F) (Boevink et al., 1998) or the plasma membrane (PM) marker GFP::SYP122 (Figures 3G–I) (Rehman et al., 2008). The reliability of RFP::NIP1;1 localization at the ER was confirmed by the ability to complement *nip1;1ko* phenotype on As(III) treatment (Figure 4).

**Figure 5:**
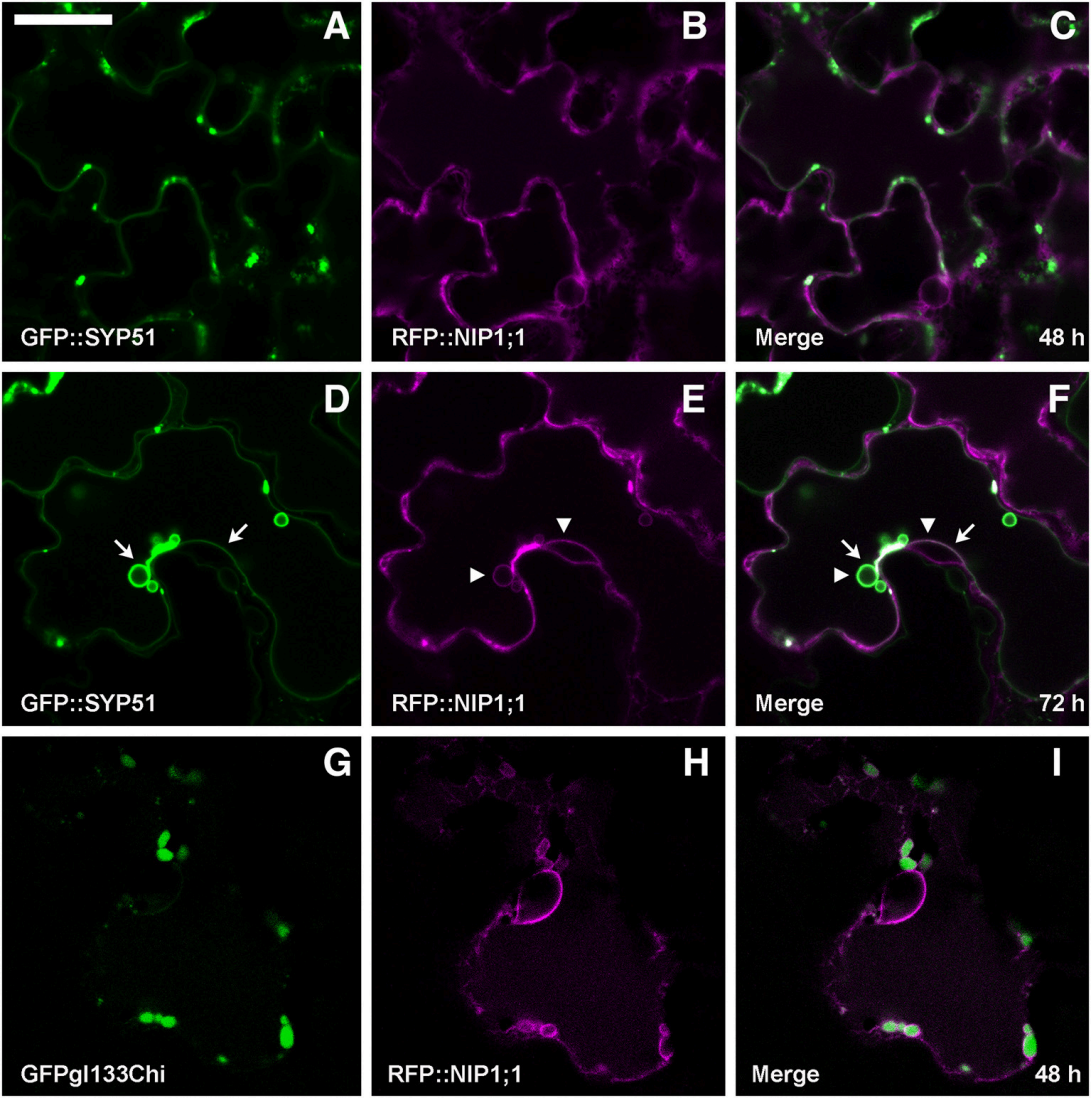
Epidermal cells' confocal images of Arabidopsis cotyledons, transiently co-expressing RFP::NIP1;1 with **(A–C)** GFP::SYP51 after 48 h of co-expression; **(D–F)** GFP::SYP51 after 72 h; **(G–I)** Golgi-independent vacuolar marker GFPgl133Chi after 48 h. The first column shows GFP signal in green, the second column shows the RFP signal in magenta, whereas the third column shows the overlay of fluorescent signals and co-localization in white. Scale bar = 20 μm.

Co-expression of RFP::NIP1;1 with GFP::SYP51 showed that the two chimerical constructs did not co-localize within the first 48 h of transient expression (Figures 5A–C). GFP::SYP51 was distributed on the tonoplast and in aggregates associated to the tonoplast while RFP::NIP1;1 clearly labeled the ER. Observations after 72 h revealed an interesting evolution of the two patterns. GFP::SYP51 induced abundant invaginations of the tonoplast (Figure 5D, arrows), producing the phenomenon often indicated as “bulbing” (Saito et al., 2002; Gattolin et al., 2009; Renna et al., 2013); while RFP::NIP1;1, still mainly on the ER (Figure 5E), was observed partially co-localized with GFP::SYP51 on the tonoplast; co-localization was particularly evident at the invagination sites (Figures 5E,F, arrowheads). The aggregates at the invagination sites appeared to have a discrete consistence (Supplementary Video 1). Even if after 48 h RFP::NIP1;1 was observed only in the ER, circular structures occasionally appeared associated to the usual reticular pattern. Since the chimera was shown not to co-localize with the Golgi marker ST::GFP, it was co-expressed with the Golgi-independent vacuolar marker GFPgl133Chi (Stigliano et al., 2013), showing that these compartments become larger during co-expression and accumulated GFPgl133Chi (Figures 5G–I). Based on these observations, we identified these compartments as the “small vacuoles” previously reported (Di Sansebastiano et al., 1998, 2001, 2007; Flückiger et al., 2003; Sanmartín et al., 2007; De Benedictis et al., 2013; Stigliano et al., 2013).

### AtSYP51/AtNIP1;1 Interaction May Be Related to Tonoplast “Bulbing”

**Figure 6:**
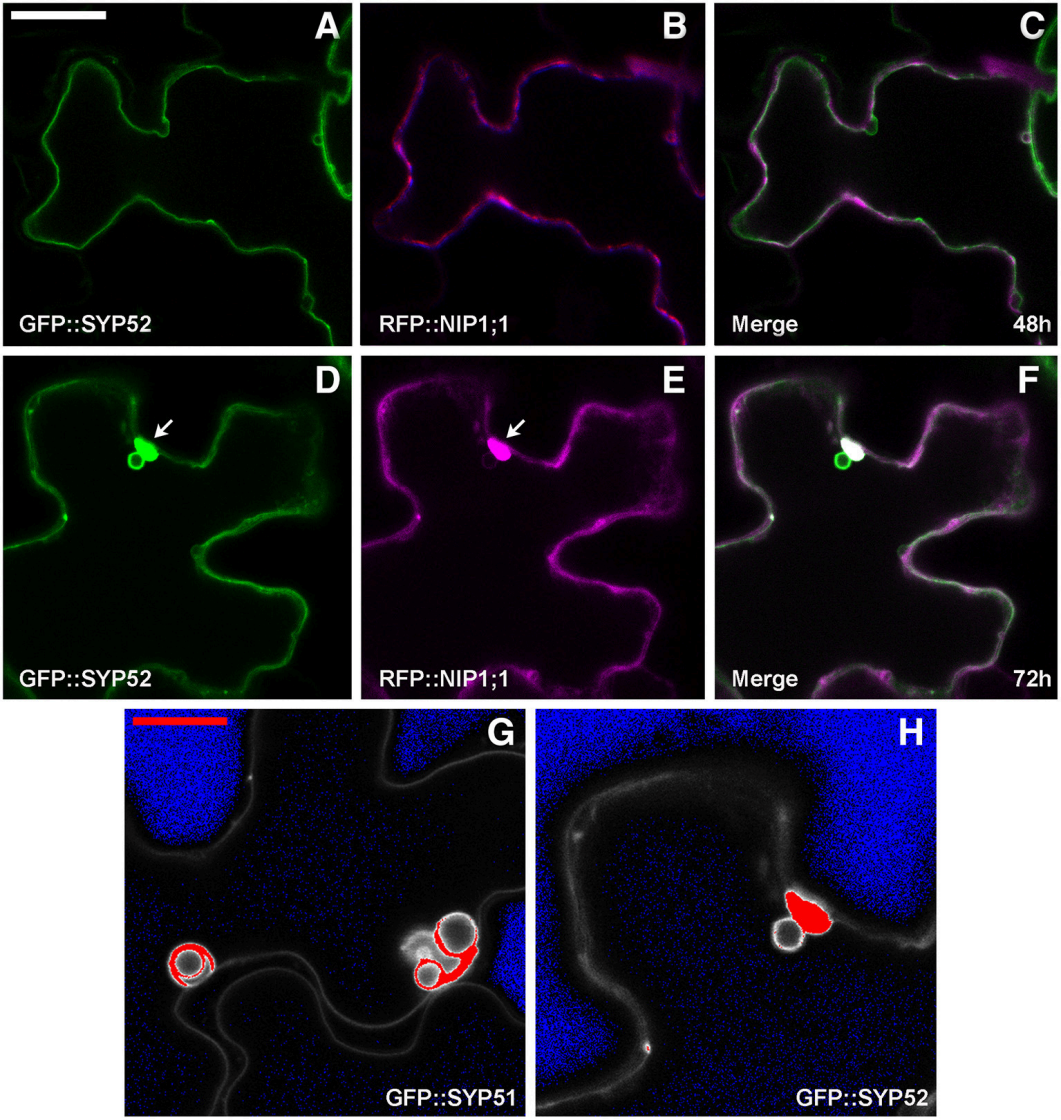
Epidermal cells' confocal images of Arabidopsis cotyledons, transiently co-expressing RFP::NIP1 with **(A–C)** GFP::SYP52 for 48 h; **(D–F)** for 72 h. The first column shows GFP signal in green, the second column shows the RFP signal in magenta whereas the third column shows the overlay of fluorescent signals and co-localization in white. **(A–F)** Scale bar = 20 μm (panel A). **(G,H)** Fluorescent signal saturation due to GFP::SYP51 **(G)** and GFP::SYP52 **(H)**. **(G,H)** Scale bar = 10 μm **(G)**.

GFP::SYP52 did not co-localize with RFP::NIP1;1, either at 48 (Figures 6A–C), or at 72 h after co-transformation (Figures 6D,E) with the exception of the aggregates observed in correspondence of the tonoplast invaginations (Figures 6D,F arrow). Moreover, the bulbing of the tonoplast was drastically less evident than when evidenced by GFP::SYP51 expression. To further investigate this difference, we analyzed the distribution of the fluorescent signal on cellular membranes. Tridimensionality of the cell and technical limits suggested adopting a very simple approach based on visualizing the maximum intensity through the indication of signal saturation (Zeiss Zen basic software). In the case of GFP::SYP52, the maximum signal intensity was observed in the aggregates associated to the tonoplast and eventually on the tonoplast itself. The intensity of signal on the invaginated membrane was always minor (Figure 6G). In the case of GFP::SYP51 the maximum signal intensity was equally distributed on all membranous districts: tonoplast, aggregates as well as bulbing invaginations (Figure 6H), relating GFP::SYP51 overexpression and accumulation with bulbing itself.

### AtSYP51 and AtSYP52 Differ in the Ability to Interact With AtNIP1;1 Through Their C-Terminal Domains

**Figure 7:**
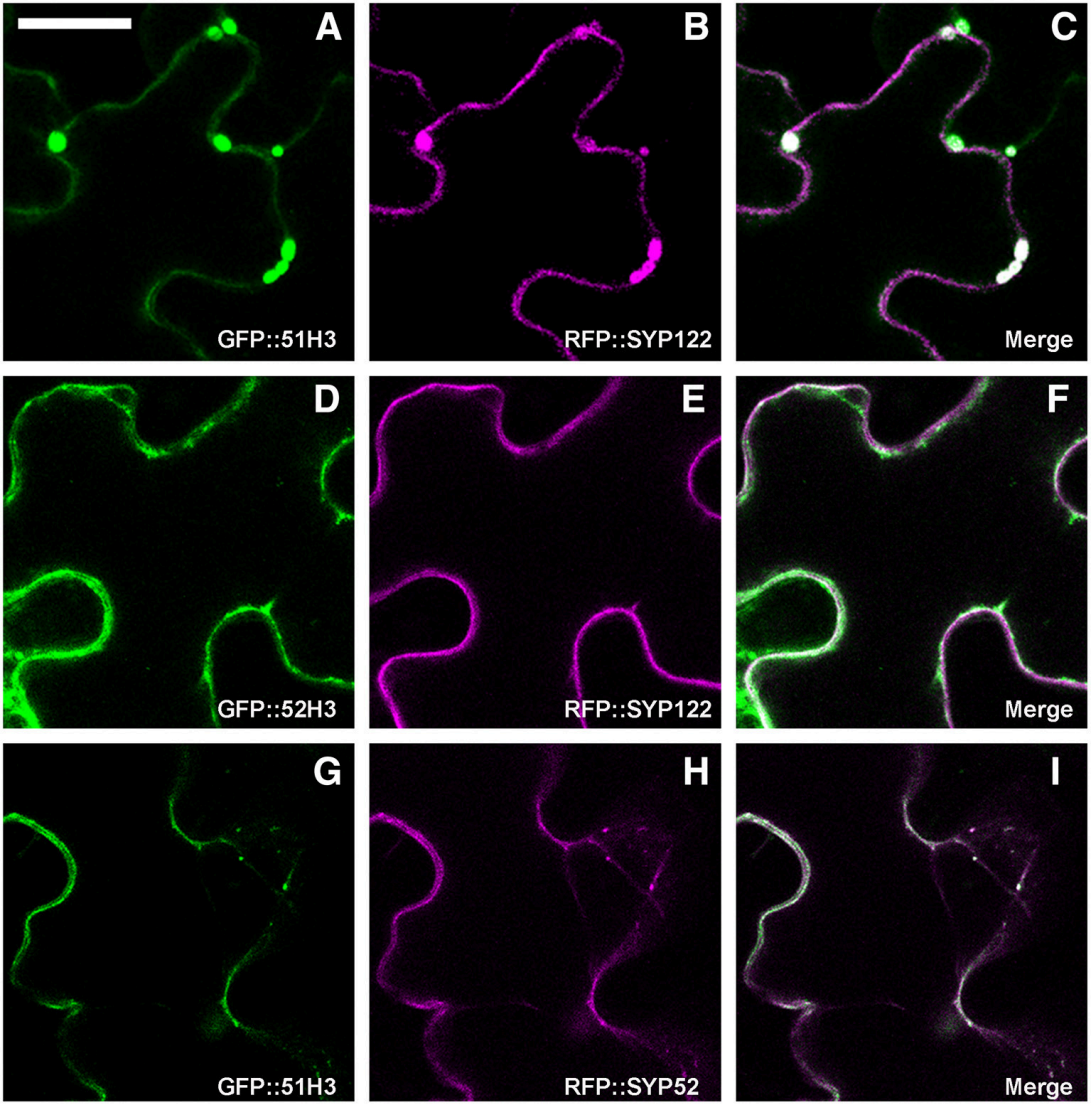
Epidermal cells' confocal images of Arabidopsis cotyledons, transiently transformed. GFP::51H3 **(A)** and RFP::SYP122F **(B)** colocalize in the MVB **(C)**; GFP::52H3 moves to TGN/EE and tonoplast **(D–F)**, not inducing MVB labeled by RFP::SYP122F **(E)**. When GFP::51H3 **(G)** and RFP::SYP52 **(H)** are co-expressed by the same cell, GFP::51H3 distribution follows that of RFP::SYP52 colocalizing **(I)** and multivesicular structures do not form. The first column shows GFP signal in green, the second column shows the RFP signal in magenta, the third column shows the overlay of fluorescent signals and co-localization in white. Scale bar = 20 μm.

It was previously shown that AtSYP51 and AtSYP52 have specific roles in the transport of vacuolar cargoes (De Benedictis et al., 2013; Faraco et al., 2013). Searching for the determinants of SNARE domains specificity, we deleted the N-terminus of the proteins, producing chimerical constructs with GFP fused to the SNARE signature domain (also called H3) and transmembrane domain of either AtSYP51 (GFP::51H3) or AtSYP52 (GFP::52H3). We observed that the two constructs had different distribution patterns. GFP::51H3 accumulated in multivesicular structures that also trapped the PM marker RFP::SYP122 (Figures 7A–C), while GFP::52H3 had a pattern similar to the native protein on dotted TGNs and tonoplast (Figures 7D–F). GFP::51H3 was accumulated in aberrant compartments as a final event of its sorting (Figure 7C). In fact when co-expressed with GFP::SYP52 (Figures 7G–I), GFP::51H3 followed the normal sorting of SYP52 (see previous figure Figures 6A–F, 7H). Increase in the rate of sorting to tonoplast during co-expression of SYP51 and SYP52 was also shown previously (De Benedictis et al., 2013).

**Figure 8:**
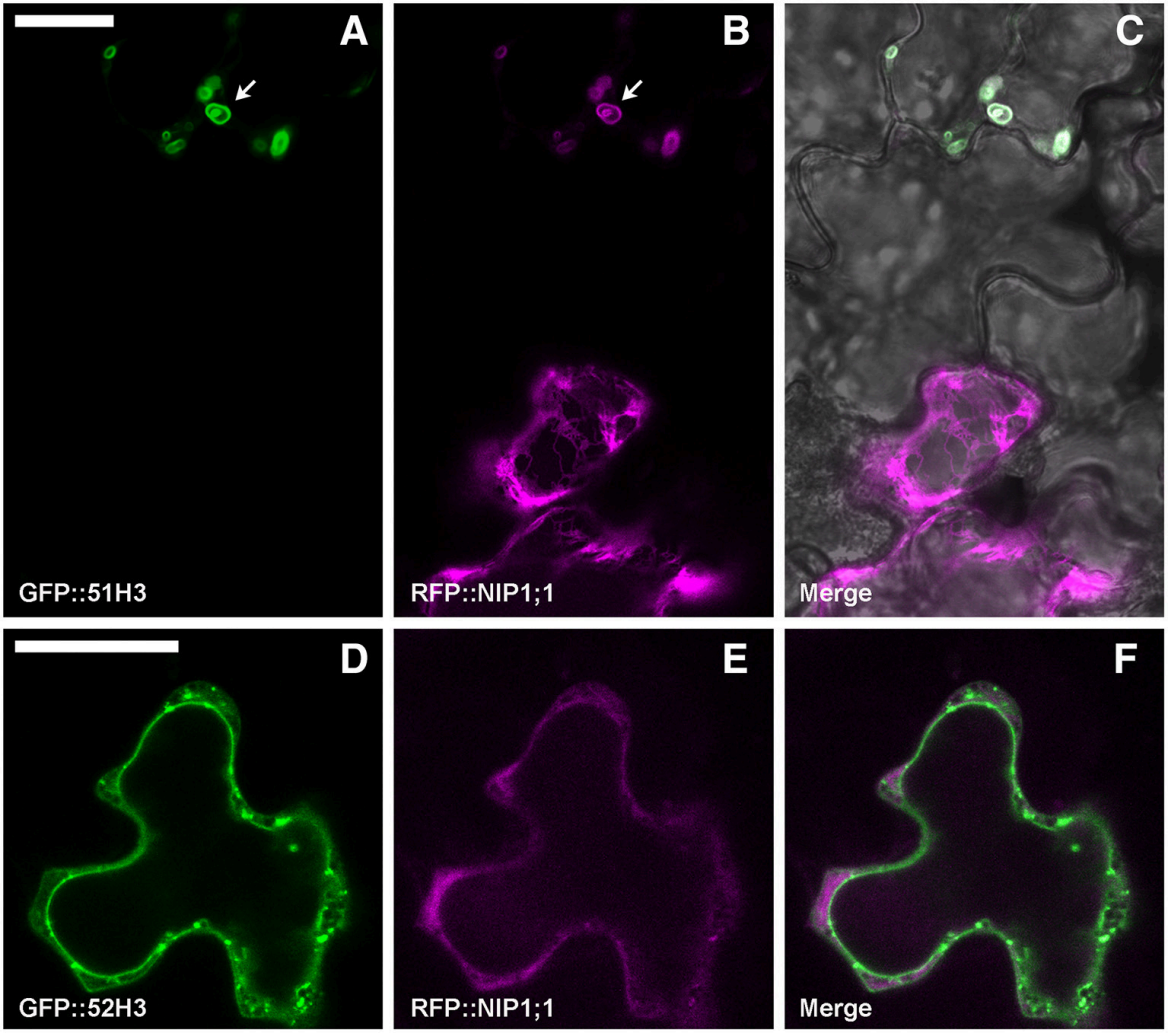
Epidermal cells' confocal images of Arabidopsis cotyledons, transiently co-transformed with RFP::NIP1;1 and **(A–C)** GFP::51H3 or **(D–F)** GFP::52H3, respectively. The first column shows GFP signal in green, the second column shows the RFP signal in magenta, while the third column shows the overlay of fluorescent signals and co-localization in white. In the case of panel **(C)**, the overlay includes transmitted light, in order to highlight that the two cells belong to the same tissue. Scale bar = 20 μm.

To test a possible interaction between NIP1;1 and the H3 variant form of SYP5, the proteins were co-expressed. *Arabidopsis thaliana* cotyledons were transformed with GFP::51H3 or GFP::52H3 and with RFP::NIP1;1. GFP::51H3 induced the formation of aberrant Multi-Vesicular Bodies (MVBs) (Figure 8A, upper cell, arrow) in which RFP::NIP1;1 was trapped and co-localized (Figure 8B, upper cell). If the cell wasn't transformed with GFP::51H3 (Figure 8A, lower cell) even if part of the same tissue (Figure 8C), RFP::NIP1;1 maintained its localization on ER (Figure 8B lower cell) even if part of the same tissue (Figure 8C). GFP::52H3, like previously showed (see previous Figures 7D–F), localized on tonoplast and dotted TGNs (Figure 8D) and RFP::NIP1;1 maintained the specific localization on ER (Figure 8E); NIP1;1 and 52H3 did not co-localize (Figure 8F).

**Figure 9:**
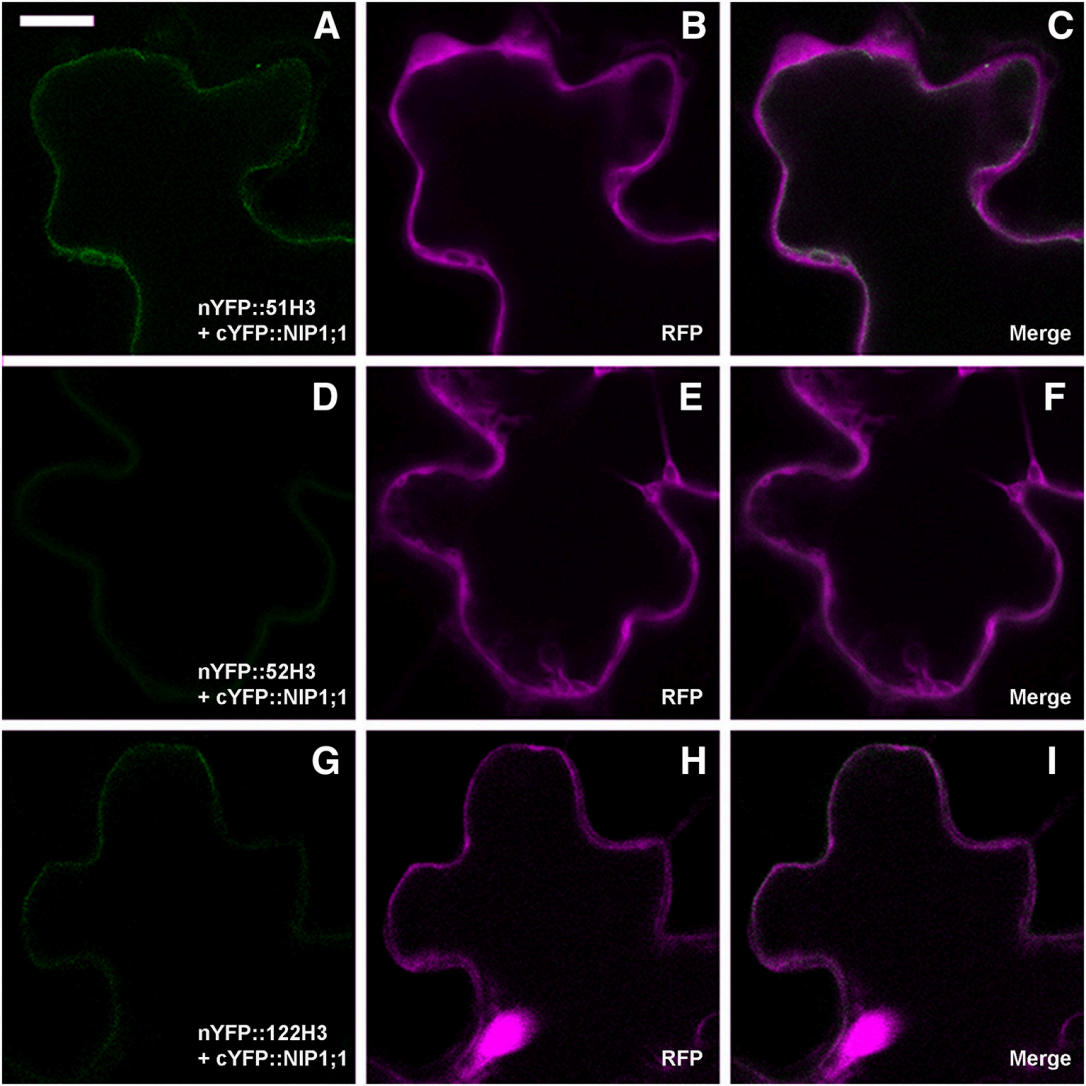
Confocal images of cotyledons' epidermal cells representative of rBiFC results for SNAREs deletion mutants. **(A)** weak interaction of nYFP::51H3 with cYFP::NIP1;1 on the tonoplast; **(B,C)** RFP clearly visible and dispersed in the cytosol; **(D)** very weak interaction of nYFP::52H3 with cYFP::NIP1;1, with no possibility to define a specific localization of the signal. **(E,F)** clearly visible RFP; **(G)** very weak interaction of nYFP::122H3 with cYFP::NIP1;1 on peripheral membrane; **(H,I)** again, well visible RFP. Scale bar = 20 μm.

To verify the persistence of the interaction *in vivo* between SNAREs H3 variant and NIP1;1, rBiFC technique (Grefen and Blatt, 2012) was applied. As(III) in the case of full length a set of controls was included. Split YFP was fused at the N-terminus of SNARE's H3 variants and the complementary part was fused alternatively to N- and C-terminus of NIP1;1. Only nYFP::51H3 showed a strong interaction with cYFP::NIP1;1 (Figures 9A–C). Surprisingly, the interaction site was again the tonoplast and not the multivesicular structures supposedly induced by nYFP::51H3. The control combinations with nYFP::52H3 **(Figures 9D–F)** and nYFP::122H3 (Figures 9G–I) showed interactions on the PM in all cases.

**Figure 10:**
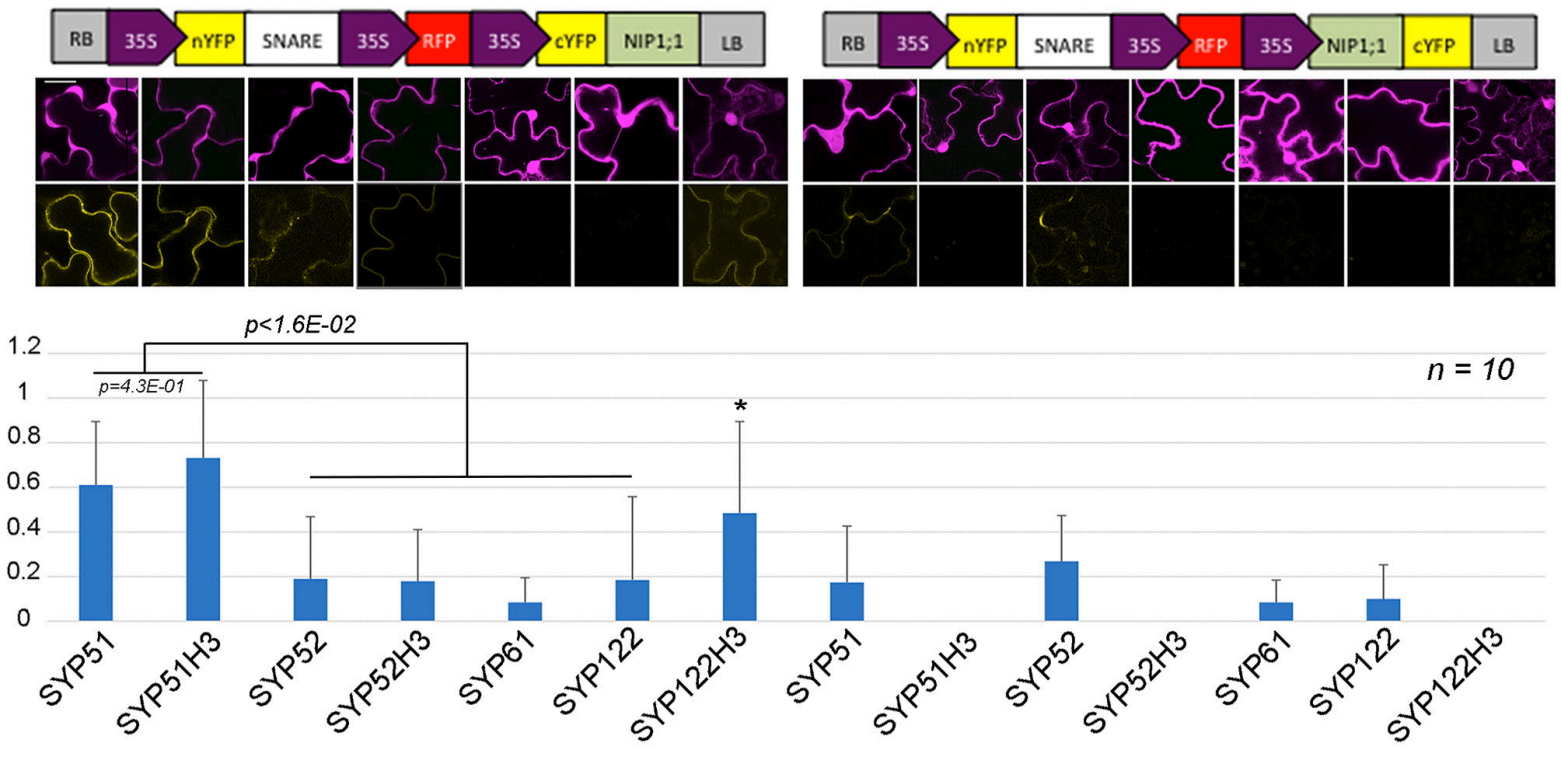
rBIFC analysis of NIP1;1 with different *A. thaliana* native and truncated SNAREs' constructs. Boxed cartoons show construct design above representative images of epidermal cells from transiently transformed *A. thaliana* cotyledons; scale bar = 20 μm. Larger confocal images are shown in Figures 2, 9. YFP/RFP mean fluorescence intensities from 10 different independent samples were calculated as the average YFP/RFP ratio. *indicates significance *p* < 1.6E–02.

In total 14 different combination of rBiFC were performed and the interactions appeared specific to the SYP51 sequences (Figure 10). When interaction signals were observed with nYFP::52H3 (Figures 9D–F) and nYFP::122H3 (Figures 9G–I), it was not localized on the tonoplast but the PM.

### AtNIP1;1 and AtSYP51 Mutants Phenotyping

To functionally characterize of NIP1;1 and SYP51, we obtained *A. thaliana* cv Columbia-0 (*Col-0*) *knock-out* lines for AtNIP1;1 (Kamiya et al., 2009) and AtSYP51 from TAIR and produced transgenic lines over expressing tagged variants of the two genes. Quantitative qPCR analysis confirmed that the TAIR lines CS68564 (*nip1;1ko)* and CS874820 (*syp51ko*) were knockouts while CS68563 was a severe knockdown for *NIP1;1* expression (Supplementary Figure 5). *Knock-out* lines were transformed with 35S driven RFP::NIP1;1 (*rfp::nip1;1*) and RFP::SYP51 (*rfp::syp51)* expression. We verified the phenotype previously described for the *nip1;1ko* mutant, documented as resistant to 15 μM As(III) (Kamiya et al., 2009; Figure 4) and complemented the phenotype with the overexpression of RFP::NIP1;1. *Syp51ko* had no evident phenotype except a 5-fold reduction of Cu(II) uptake (Supplementary Figure 6). To test overexpression effects and at the same time validate the functionality of tagged proteins, transgenic lines of *A. thaliana* cv Col-0 were produced and selected as homozygous lines expressing RFP::NIP1;1 and RFP::SYP51. The fluorescent proteins analyzed by immunoblotting showed to have the correct expected molecular weight when associated to the membranous cellular fraction but also showed to release RFP in the soluble fraction because of degradation (see previous Supplementary Figure 2). The localization shown after transient expression was confirmed for both proteins (see previous Supplementary Figures 3, 4) and the additional fluorescence in the vacuole lumen was evidently due to tag proteolysis. A more detailed analysis of metals and metalloids uptake capacities was then performed.

### AtNIP1;1 Role in As(III) Translocation

NIP1;1 is known to transport As(III) and Sb(III) (Kamiya and Fujiwara, 2009; Kamiya et al., 2009) but a recent work reported the uptake of As(III) also in *nip1;1ko* lines (Ji et al., 2017). We also observed As(III) uptake (2% less than control) in *nip1;1ko* while testing uptake of different elements in mutant lines (Supplementary Figure 6). The mutant *nip1;1ko* showed an increase of Zn(II) and a 20% reduction of Sb(III) but As(III) uptake appeared normal; *rfp::nip1;1* mutant showed the most altered behavior with reduced Cu(II) and increased Zn(II) and Sb(III) uptake;, As(III) uptake increase was moderate (19%). These observations supported that permeability was unlikely to be the explanation of the *nip1;1ko* resistance to As(III).

**Figure 11:**
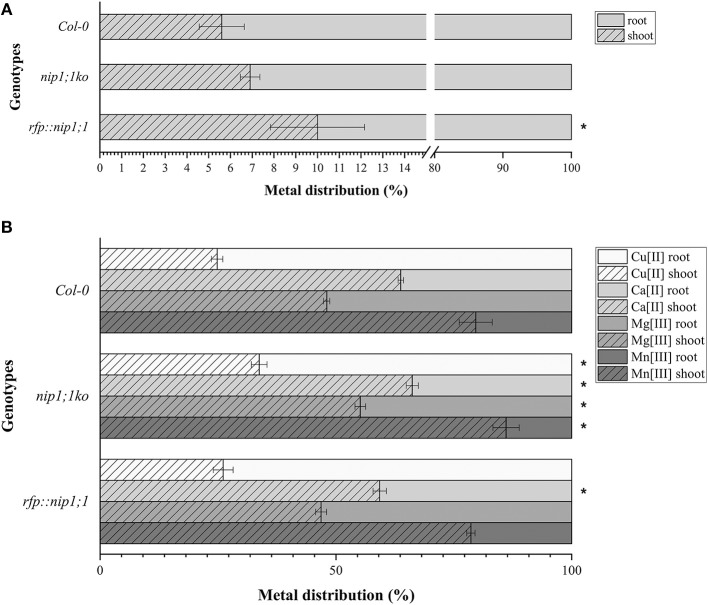
Distribution, measured by ICP-AES of **(A)**. AS (significance (*) expressed as *p* = 0.024) and **(B)** Ca, Cu, Mg, and Mn in the plants: the percentage of metals in the root resulting from direct uptake and the percentage in the shoot due to translocation (significance (*) of difference with Col0 control expressed as *p* = 0.041). *n* = 5.

The mutant syp*51;1ko* showed a characteristic reduction of Cu(II) uptake (80% less than control) in addition to other small alterations. Also, the RFP::syp*51* line showed a reduction of Zn(II), As(III) and Sb(III) uptake (Supplementary Figure 6). These phenotypes did not clarify the relation between SYP51 and NIP1;1, but they made apparent that As(III) uptake is not preferentially due to NIP1;1. We therefore hypothesize that translocation plays a role in the As(III) tolerance exhibited by the *nip1;1ko* mutant. The evaluation of translocation over a period of 7 days was performed on *Col0, nip1;1ko*, and *RFP::nip1;1* plants. Surprisingly, translocation activity was always observed, and even increased in *nip1;1ko* (Figure 11A). To assess whether other metals' symplastic transport was differently altered in different genotypes, the translocation of Ca, Cu, Mg, and Mn normally present in the medium was analyzed. The translocation showed to be all increased in the *nip1;1ko* but not in *rfp::nip1;1* (Figure 11B), showing that a transport alteration was due to NIP1;1 deficiency.

## Discussion

### Traffic Mechanism Features

A key feature of membrane traffic is the specificity in the membrane fusion reaction. The soluble N-ethylmaleimide-sensitive factor attachment protein receptor (SNARE) proteins are essential specificity determinants, but found to be expressed in the plant cell by redundant gene families that pose evident problems in their characterization through knock-out mutants and require alternative approaches (Di Sansebastiano et al., 2009). We focused on SYP5 gene family members interactome, specifically on aquaporins (AQP) because both SNAREs and AQP appear more abundant than required by their well-characterized cellular function, and may have additional secondary functions not yet characterized. SYP51 and the closely related homolog SYP52 have already shown to display distinguishable characteristics (De Benedictis et al., 2013). The discovery of different specific functions for the members of a single SNARE's gene family appears to be related to the evolution of complex cellular processes in eukaryotes. AQPs are emerging as potential interactors of other SNAREs: SYP121 physically interact with PIP2;5 (Besserer et al., 2012) and both SYP121 and SYP61 interacts with PIP2;7 (Hachez et al., 2014).

In our study, we suggest that there are many more potential AQP-SNARE interactions than expected. The BAR database analysis revealed interaction between AQPs and PM QaSNARE SYP132 (Arabidopsis Interactome Mapping Consortium, 2011; Mukhtar et al., 2011), Golgi Qc-SNAREs BET12 (Hermjakob et al., 2004; Toufighi et al., 2005; Arabidopsis Interactome Mapping Consortium, 2011; Jones et al., 2014) and Golgi Qc-SANRE SFT12 (Hermjakob et al., 2004; Toufighi et al., 2005; Jones et al., 2014). Moreover, 11 out of 15 SNAREs interacting with AQPs, interact with NIP1;1. Several of the interacting SNAREs are Qc-SNAREs. Fifteen out of the eighteen interacting AQPs putatively interact with Qc-SNAREs. Some AQPs like NIP1;1 are predicted to interact with several Qc-SNAREs and among these NIP1;1 is predicted to interact with the Qc-SNARE SYP51 but not with the closely related Qc-SNARE SYP52.

A SYP51/NIP1;1 interaction *in vivo* was tested using the technique of Ratiometric Bimolecular Fluorescence complementation (rBiFC) (Grefen and Blatt, 2012). This method allows a clear evaluation of interaction efficiency since the reconstituted signal can be compared with the expression of cytosolic RFP to be used as a reference (Xing et al., 2016, 2017). In addition to the control provided by the internal reference, we planned a number of controls to ensure the significance of the interaction (Figure 10). In fact, to test the specific interaction of SYP51 with NIP1;1 we also prepared control reactions with the very similar homolog Qc-SNARE SYP52 (De Benedictis et al., 2013), the similar but more distant SYP61 (Ebine et al., 2008) and the unrelated QaSNARE SYP122 (Rehman et al., 2008). Only nYFP::SYP51 strongly interacted with cYFP::NIP1;1, confirming the putative interaction discovered *in silico* and showing it was relatively specific.

The interaction was observed on tonoplast also when SYP51H3 mutant was used. In this case, the total average fluorescence evidenced over several independent experiments was higher than that calculated for native SYP51. In general rBiFC with native SYP51 showed stronger signal than rBiFC with SYP51H3 (see previous Figures 2, 9, 10) but cells showing “donut-like structures” had reduced amount of average fluorescence and reduced the average value. In rBiFC with SYP51H3 “donut-like structures” were not observed and for this reason the total YFP/RFP mean fluorescence ratio appeared higher (Figure 10).

Interestingly, neither the tonoplast nor the small aggregates and “donut-like structures” fully corresponded to interactors' known localization. GFP/RFP::SYP51 is normally distributed on tonoplast but also in dots and aggregates previously shown to be related to TGN (De Benedictis et al., 2013) and on large invaginations of tonoplast inside the vacuole (the so-called vacuole bulbing); GFP/RFP::NIP1;1 appears restricted to the ER. The interaction on the tonoplast seems then to be very specific and not simply related to co-localization. GFP/RFP::NIP1;1 was found distributed in the ER and not in the PM as previously reported by Kamiya and Fujiwara (2009), Kamiya et al. (2009). A more recent work reported again the localization on the PM but the fusion with the tag was C-terminal (Ji et al., 2017), the same orientation that in our experiments produced a cytosolic distribution. Moreover, the imaging reported in both studies did not present any co-localization evidence. Here we show co-localization with the well-known ER marker GFP::KDEL and absence of colocalization with Golgi apparatus marker ST::GFP or PM marker GFP::SYP122. Despite the lack of colocalization with Golgi stacks, RFP::NIP1;1 appeared to occasionally reach the vacuole. In fact we observed a partial co-localization with the tonoplast marker GFP::SYP51 (De Benedictis et al., 2013). Interestingly RFP::NIP1;1 was never observed on tonoplast when other markers were co-expressed. Evidently GFP::SYP51 overexpression was sufficient to relocate a small part of RFP::NIP1;1 from ER to tonoplast. An additional observation relating RFP::NIP1;1 to vacuoles was its partial colocalization with the vacuolar marker GFPgl133Chi (Flückiger et al., 2003; Stigliano et al., 2013). RFP::NIP1;1 labeled the membrane of small vacuoles whose lumen was occupied by GFPgl133Chi. These small vacuoles were also occasionally observed in the absence of GFPgl133Chi overexpression but, of course, they were more evident in the presence of the soluble marker. In all cases, the small vacuoles appeared connected to the ER and gave the impression to be directly generated from it.

Since RFP::NIP1;1 was never observed co-localized with Golgi marker, we hypothesized that it reaches the tonoplast through a direct ER-to-vacuole route bypassing the Golgi as shown for GFPgl133Chi (Stigliano et al., 2013). RFP::NIP1;1 labeled ER membrane can then reach tonoplast through a Golgi independent route and interaction with SYP51 can occur on this membrane. Co-localization is anyhow not sufficient to promote interaction. We show that using deletion mutants of SYP5s, called H3, we cause a jam of membrane traffic that induce RFP tagged NIP1;1 to colocalize with the construct GFP::51H3 in multivesicular compartments but the interaction revealed by rBIFC did not occur in such aberrant compartment but remained specific and localized on the tonoplast. H3 variants of several SNAREs represent additional controls and demonstrate that the H3 domain of any SNARE, deprived of the regulatory N-terminal domain, have more affinity for NIP1;1 but the interaction did not depend uniquely on co-localization. In fact, we saw the GFP::51H3 and RFP::NIP1;1 colocalize in multivesicular compartments but the only interaction was observed between nYFP::51H3 and cYFP::NIP1;1 on the tonoplast **(**see previous Figures 9A–C) and not in the multi vesicular compartments.

In addition to the tonoplast, strong interaction between nYFP::SYP51 and cYFP::NIP1;1 was observed in small compartments that appeared similar to “donut-like structures” described also in other studies (Jaillais et al., 2006; Honig et al., 2012). Their occurrence, limited to few cells and with regular shape and dimensions, suggests these are different from the so-called OSER (Snapp et al., 2003). Even if related to aberrant behavior due to overexpression of proteins, their formation remains an interesting phenomenon. These structures were clearly different from multivesicular compartments induced by the H3 mutants. They were often labeled by GFP::SYP51 alone, even if they were hardly noticeable because often masked by the abundant tonoplast bulbing induced by the same marker. Similar structures were also labeled by RFP::NIP1;1 when co-expressed with GFPgl133Chi (see previous Figure 5H). Since we did not observe NIP1;1 colocalizing with Golgi marker (see previous Figure 3F) but we know that SYP51 sorting, instead, is dependent on Golgi, we hypothesize that the interaction between NIP1;1 and SYP51 occurred in a post-Golgi compartment related to TGN, which may represent a regulatory cross-talk between conventional and “unconventional” vacuolar sorting pathways.

The balance between the two kinds of proteins and the consequent extension of the interaction could influence post-Golgi compartments architecture possibly contributing to regulate different vacuolar sorting machineries involved in plant cell development and adaptation (Di Sansebastiano et al., 2014). The regulation of these compartments may produce the rich variability of post-Golgi compartment (MVBs, MASCs, neutral vacuoles) still demanding full characterization. The existence of this kind of interaction, possibly very variable, may influence the sub-compartmentalization of TGN-related compartments, as for example those characterized by RABA2a, RABA4b, or RABA1, and contribute to the challenges of confirming the mechanisms involved to confirm the mechanisms involved (Rosquete et al., 2018).

We found very interesting that an rBiFC signal was detected on the PM with SYP52H3 and SYP122H3. The signal was weak but suggested that NIP1;1 could also reach the PM.

We suggest here a new mechanism to regulate the proportions between direct ER-to-vacuole transport and Golgi sorting. It is based on the interaction of NIP1;1 and SYP51 on the tonoplast and possibly on TGN-derived compartments. SYP51 is normally poorly expressed (De Benedictis et al., 2013) and the observation of abundant invaginations when overexpressed, inducing the so called “vacuole bulbing,” may be indicated as a specific effect. Here we show that, confirming a specific role for SYP51, the very closely related SNARE SYP52 does not induce “bulbing.”

If SYP51 excess on the tonoplast lead to tonoplast invagination and recycling, it is reasonable to hypothesize that it is part of a control mechanism. Most of tonoplast membrane proteins reach their destination via a Golgi-independent route (Pedrazzini et al., 2016) and the excess of SYP51 may be defined when its arrival through the Golgi is in excess compared to new membrane arriving from the ER. Interactions with ER-derived proteins may keep SYP51 under control when not engaged in SNARE-complexes. Previously we already evidenced that SYP51 reaching the tonoplast causes traffic interferences that evidence a double function. SYP51 works as a tSNARE on TGN and as an iSNARE on tonoplast (De Benedictis et al., 2013). The very specific interaction with NIP1;1 could provide the control mechanism sensing disproportioned traffic. We see NIP1;1 reaching the tonoplast only when SYP51 is overexpressed but it doesn't normally reach this membrane. Since we observed strong interaction also in post-Golgi small compartments we suggest a new event of ER-to-vacuole traffic with the involvement of TGN, as previously shown for AtRMRs by Occhialini et al. (2016, 2018) and Di Sansebastiano et al. (2017). Although we cannot exclude that an accelerated translocation of the protein through the Golgi could make the signal not apparent in this organelle.

Hundreds of TGN buds from trans-Golgi, and some may contribute to this new cross-talk between the Golgi-dependent and unconventional Golgi-independent traffic (for a review on traffic see Di Sansebastiano et al., 2017; for a review on TGN see Rosquete et al., 2018). The interaction takes place downstream of the TGN itself because the interference of the H3 mutants induce the formation of MVB as typically formed from TGN membranes, but these compartments do not allow interaction. The GFP::51H3 induced MVBs trap the ER protein RFP::NIP1;1 so it is reasonable to suppose that ER membrane came in contact with TGN membrane before maturing in a form where SYP51 and NIP1;1 can interact. This downstream compartment may correspond to the pre-vacuolar compartment (PVC) but still be different by late PVC (LPVC), which is a matured version of PVC, depleted for the vacuolar sorting receptors (VSRs; Foresti et al., 2010).

### NIP1;1 Function Is Important for As(III) Transport

The localization of NIP1;1 in the ER membrane is incoherent with As(III) uptake. In fact, in this study, as in a previous work (Ji et al., 2017) *nip1;1ko* lines regularly uptake As(III). It was predictable that As(III) uptake was not relying on a single mechanism since other examples can be cited. In rice and arabidopsis roots the uptake of As(III) occurs via two mechanisms (Li et al., 2016). The first transport route involves As(V) translocation from soil to aerial parts of the plants and occurs through high affinity phosphate transporter (PT). The second route sees As(III) uptake by aquaporins. In rice, As(III) enters through Lsi1 (OsNIP2;1), while Lsi2 mediates As(III) efflux to the xylem (Ma et al., 2008). The two routes may be closely interconnected. The expression patterns of Zea mays NIP1;1 correlates with phosphate transporters PHT4;2 and PHT4;6 as well as with chloride channel protein CLC-f, NIP2;1 correlates with phosphate transporters PHT5, while CLC-d displays the same expression patterns as ZmNIP2;2 (Yue et al., 2012).

If *nip1;1ko* has no effect on As(III) uptake, but the mutant is nonetheless resistant to the metalloid, then translocation or compartmentalization of the metalloid may be involved in a tolerance mechanism. In our study we see that, even if both uptake and translocation are not impaired in the *nip1;1ko* genotype, other elements' translocation is increased, indicating an alteration of other transporters' expression and/or activity. Indeed also *syp51ko* and overexpressing lines *rfp::nip1;1* and *rfp::syp51* alter other elements' uptake and confirm that these proteins play a role in metals and metalloids homeostasis.

Aquaporins evolved modifying both specificity of their expression pattern and water permeation properties, maintaining the ability to be differentially induced. So PHTs and CLC-d correlate to certain maize AQPs (Yue et al., 2012) NIP1;1 overexpression appears to enhance Zn(II) uptake but also increase As(III) translocation to aerial parts. Translocation relies on intracellular homeostasis and cell-to-cell communication through the ER. We suggest that NIP1;1 can increase translocation of As(III), coherently with its localization in the ER, but it has a prominent role in intracellular compartmentalization so that its absence alters the vacuole characteristics and, as a consequence, As(III) tolerance. The different involvement of vacuolar traffic machineries in homeostasis and detoxification processes and the connections with autophagy have been previously studied (Kulich and Žárský, 2014; Sharma et al., 2016; Barozzi et al., 2017; Faraco et al., 2017; Ariani et al., 2018; Liu et al., 2018).

We observed that Cu(II) uptake is sensibly reduced in the *rfp::nip1;1* and in the *syp51ko* suggesting the possibility of some kind of correlation between the relative proportions of the two proteins. Zn(II) uptake was strongly increased by *rfp::nip1;1* but also moderately increased by *nip1;1ko* and *syp51ko*. Further studies are required to test the relationships between variation in membrane trafficking and SYP51-NIP1;1 interactions.

## Material and Methods

### *In silico* Interactions Network Analysis

*In silico* analysis was performed interrogating the BAR database (Geisler-Lee et al., 2007) using the software Cytoscape 3.4.0 (Shannon et al., 2003). It is an open-source program (http://www.cytoscape.org/download.php), that connects to several online databases in order to create and graphically edit an interaction network.

The following proteins were used as initial input: SYP111 (At1g08560), SYP112 (At2g18260), SYP121 (At3g11820), SYP122 (At3g52400), SYP123 (At4g03330), SYP124 (At1g61290), SYP125 (At1g11250), SYP131 (At3g03800), SYP132 (At5g08080), SYP21 (At5g16830), SYP22 (At5g46860), SYP23 (At4g17730), SYP31 (At5g05760), SYP32 (At3g24350), SYP41 (At5g26980), SYP42 (At4g02195), SYP43 (At3g05710), SYP81 (At1g51740), VTI11 (At5g39510), VTI12 (At1g26670), VTI13 (At3g29100), VTI14 (At5g39630), GOS11 (At1g15880), GOS12 (At2g45200), MEMB11 (At2g36900), MEMB12 (At5g50440), NPSN11 (At2g35190), NPSN12 (At1g48240), NPSN13 (At3g17440), SEC20 (At3g24315), SEC221 (At1g11890), SEC222 (At5g52270), BET11 (At3g58170), BET12 (At4g14455), SYP51 (At1g16240), SYP52 (At1g79590), SYP61 (At1g28490), SYP71 (At3g09740), SYP72 (At3g45280), SYP73 (At3g61450), SFT11 (At4g14600), SFT12 (At1g29060), USE11 (At1g54110), USE12 (At3g55600), SNAP29 (At5g07880), SNAP30 (At1g13890), SNAP33 (At5g61210), VAMP711 (At4g32150), VAMP712 (At2g25340), VAMP713 (At5g11150), VAMP714 (At5g22360), VAMP721 (At1g04750), VAMP722 (At2g33120), VAMP723 (At2g33110), VAMP724 (At4g15780), VAMP725 (At2g32670), VAMP726 (At1g04760), VAMP727 (At3g54300), YKT61 (At5g58060), YKT62 (At5g58180).

The emerged interactome revealed the interaction between SYP51 and NIP1;1, a smaller network was then produced including only the interactors of SYP51, SYP52 and NIP1;1. For control purposes the network was expanded with the SYP61 interacting SNAREs and AQPs and produced a network of 84 interactors (Supplementary Table 1).

### Plasmid Constructs

CDS from existing templates of AtSYP51 (De Benedictis et al., 2013) and its deletion mutant 51H3 (Di Sansebastiano et al., 2015), AtSYP52 (De Benedictis et al., 2013) and its deletion mutant 52H3 (Barozzi, 2018), AtSYP122 (Rehman et al., 2008), AtNIP1;1 (AT4g19030), GFPgl133Chi (Stigliano et al., 2013), GFP::KDEL (Nesler et al., 2017), Rat Sialyltransferase firt 53aa (Saint-Jore-Dupas et al., 2006) were amplified (Supplementary Table 2) and cloned, using Gateway system (Life Technologies) in pDEST: pK7WGF2 for GFP fusion in N-terminus (GFP::SYP51; GFP::51H3; GFP::SYP52; GFP::52H3; GFP::SYP122; GFP::NIP1;1); pK7FWG2 for GFP fusion in C-terminus (NIP1;1::GFP; ST::GFP); pH7WGR2 for RFP fusion in N-terminus (RFP::SYP51; RFP::SYP52; RFP::SYP122; RFP::NIP1;1); pK2GW7 for cloning without tagging (GFPgl133Chi; GFP::KDEL).

### *In vivo* Interaction Assay With Ratiometric Bimolecular Fluorescence Complementation (rBiFC)

CDS of AtSYP51 and its deletion mutant 51H3, AtSYP52 and its deletion mutant 53H3, AtSYP122 and its deletion mutant 122H3, AtSYP61 and AtNIP1;1 were amplified (Supplementary Table 2) from existing templates and cloned, using Gateway system (Life Technologies), into pBiFCt-2in1_NC or NN allowing for (i) simultaneous cloning of two genes into the same vector backbone and (ii) ratiometric analysis due to additional expression of mRFP (Grefen and Blatt, 2012; Xing et al., 2017). Fluorescence intensity (reconstituted YFP vs. RFP) was measured after transient Agrobacterium-mediated transformation of *A. thaliana* seedlings. Several tens of cells were observed; 10 images were acquired with similar settings to perform statistics reported in Figure 10 (*n* = 10).

### Plant Material and Growth Conditions and Transformation Methods

*Arabidopsis thaliana* (ecotype Colombia) seeds (about 30/experiment), vernalized at 4 °C in the dark for 24 h, were germinated on modified MS (Murashige and Skoog, 1962) salts 1.1 g/L, sucrose 10 g/L, agar 8 g/L) and grown 5–6 days. They were soaked in the same petri dish with 10 mL co-cultivation medium (modified MS without agar, Acetosyringone 0.1 mM Silwet L-77 0.005%) supplemented with exponentially grown *A. tumefaciens* cells at final density of OD_600_ = 0.5 (6 × 10^8^ cfu/mL). Co-cultivation was carried out in darkness at the same temperature as seedling growth for 48–72 h before microscopic observation (Li and Nebenführ, 2010; Lionetti et al., 2017).

### Transgenic Plants

Stably transformed *A. thaliana* plants expressing *rfp::nip1;1* and *rfp::syp51* were generated by floral dipping and *Agrobacterium*-mediated transformation of corresponding plasmids into *nip1;1ko* (CS68564) and wild-type Col-0 plants (Clough and Bent, 1998). Transformed Arabidopsis seeds were selected on MS (Murashige and Skoog, 1962) growth medium supplied with hygromycin (100 mg/l). The subcellular distribution of fluorescent proteins appeared identical in each of five independent lines per transformation and the lines with the strongest expression was selected for further experiments. To confirm the expression of genes in transgenic plants, RNA and proteins were analyzed (Renna et al., 2013; KO lines RNA analysis shown in Supplementary Figure 2).

### Confocal Microscopy

Transiently and stably transformed Arabidopsis plants were examined using a confocal laser scanning microscope LSM 710 Zeiss (ZEN Software, GmbH, Germany) mounting material in water (Grieco et al., 1999). GFP was detected within the short 505–530 nm wavelength range, assigning the green color, RFP within 560–615 nm assigning the red color. Excitation wavelengths of 488 and 543 nm were used. The laser power was set to a minimum and appropriate controls were made to ensure there was no bleed-through from one channel to the other. Distribution of each fluorescent protein was observed in no < 5 independent replicates from temporally independent experiments. Images were processed using Adobe Photoshop 7.0 software (Mountain View, CA, USA).

### Root Growth Assay Under Stress

Sterile *A. thaliana* seeds, vernalized at 4°C in the dark for 24 h were germinated on petri dishes placed vertically containing modified MS (2.2 g/L salts, 1% sucrose, 1% agar) at 21°C, light cycle 16 h (100 μE). Four days after germination seedlings dimensionally homogeneous where moved to new plates containing As^3+^ (NaAsO_2_ 15 μM). The starting point of the root was labeled manually with a marker and elongation was calculated 6 days later as an index normalized to the average elongation of the control plants. Fifty plants were tested in 5 different replicates. The *p* values were obtained using two samples *t*-test function integrated in the OriginPro 2017 suite.

### Metals Uptake and Translocation Assays

Sterile *A. thaliana* seeds of all genotypes, vernalized at 4°C in the dark for 24 h were germinated on Petri dishes placed vertically at 21°C, light cycle 16 h (100 μE). Fourteen days after germination plantlets were moved in multi-well plates (12 wells) containing 3 mL modified liquid MS (2.2 g/L salts, 1% sucrose) as control or supplemented with Cu^2+^ (CuSO_4_ 20 μM), Zn^2+^ (ZnSO_4_ 200 μM), As^3+^ (NaAsO_2_ 20 μM), or Sb^3+^ (Sb2O3 20 μM).

Each experimental replica was constituted by two multi-well plates placed, one at 21°C and the other at 4°C (placed on ice under the same light source of the first) and incubated 6 h. Plantlets were then washed once with a chelating solution (20 μM EDTA in water; pH 8.5) and three times in water, dried and desiccated at 60°C. After the measure of the dry weight samples were mineralized in according to Huang and Schulte (Huang and Schulte, 1985) with some modification; the sample were moved to 1 mL HNO_3_ (ICP grade) at 100°C for 15 min. Then 1 mL H_2_O_2_ (ICP grade) was added and the samples were incubated at 100°C for 2 h. At the end of mineralization volume was adjusted to 10 mL with ICP grade water and analyzed using ICP/AES.

Cu, Zn, As, and Sb values were converted in ppm dividing the mg/L read by dry weight and multiplying by the final volume. For each genotype, the ppm value at 4°C in control conditions has been subtracted to ppm values of treatments at 4°C producing the value Δ4°C that represents the variation (if any) in passive uptake. The same was done for samples treated at 21°C, producing the value Δ21°C representing the total uptake by metabolically active plants. The difference between values Δ4°C and Δ21°C, normalized to the controls, represented the effect on active plant metals uptake (Barozzi, 2018). Number of replicates is indicated in the corresponding figure. The *p* values were obtained using two samples t test function integrated in the OriginPro 2017 suite.

## Author Contributions

FaB, G-PD, and PP designed the research and analyzed the data. G-PD wrote the first draft of the manuscript. FaB and PP wrote sections of the manuscript. FaB, G-PD, PP, GS, DM, and LR performed the experiments. FeB, GP, and FF contributed to manuscript revision. All authors read and approved the submitted version.

### Conflict of Interest Statement

The authors declare that the research was conducted in the absence of any commercial or financial relationships that could be construed as a potential conflict of interest.
