# Supplementary material for: Variation in Membrane Trafficking Linked to SNARE AtSYP51 Interaction With Aquaporin NIP1;1
Source: Front Plant Sci. 2019 Jan 9;9:1949. doi: 10.3389/fpls.2018.01949 (PMC6334215; doi:10.3389/fpls.2018.01949)
Supplement: Supplementary file 2 [file Data_Sheet_1.pdf]

Supplementary Material

Variation in membrane trafficking linked to SNARE AtSYP51 interaction with aquaporin NIP1;1

Fabrizio Barozzi[1], Paride Papadia[2]*, Giovanni Stefano[3], Luciana Renna[3], Federica Brandizzi[3], Danilo Migoni[2], Francesco Paolo Fanizzi[2], Gabriella Piro[1], Gian-Pietro Di Sansebastiano[1]*

1- Laboratory of Botany, DISTEBA (Diartimento di scienze e tecnologie biologiche e ambientali), University of Salento, Lecce, Italy
2- Laboratory of General and Inorganic Chemistry, DISTEBA (Diartimento di scienze e tecnologie biologiche e ambientali), University of Salento, Lecce, Italy
3- MSU DOE-Plant Biology Lab, Michigan State University, East Lansing, USA, Michigan

* Correspondence: Prof. Gian-Pietro Di Sansebastiano, gp.disansebastiano@unisalento.it ; Prof. Paride Papadia, paride.papadia@unisalento.it.

Supplementary Tables

Name	Tair accession number	Function
SYP121	At3g11820	SNAP receptor activity, protein binding, SNARE binding, protein membrane anchor.
TIP2;2	At4g17340	water channel activity.
At5g01430	At5g01430	molecular_function_unknown.
KCO1	At5g55630	outward rectifier potassium channel activity, ion channel activity, metal ion binding, calcium-activated potassium channel activity, potassium ion leak channel activity.
MAN1	At3g08040	transporter activity, antiporter activity, citrate transmembrane transporter activity, drug transmembrane transporter activity, metal ion transmembrane transporter activity.
NPSN12	At1g48240	SNAP receptor activity, SNARE binding, protein transporter activity.
At4g38250	At4g38250	amino acid transmembrane transporter activity, neutral amino acid transmembrane transporter activity.
TIP2;1	At3g16240	water channel activity, identical protein binding, methylammonium transmembrane transporter activity, urea transmembrane transporter activity, ammonium transmembrane transporter activity.
At1g64150	At1g64150	magnesium ion transmembrane transporter activity, manganese ion transmembrane transporter activity.
UMAMIT41	At3g28050	transmembrane transporter activity.
At4g35080	At4g35080	nickel cation transmembrane transporter activity.
SYP132	At5g08080	SNAP receptor activity, SNARE binding
PIP2;8	At2g16850	water channel activity, protein binding.
UMAMIT33	At4g28040	transmembrane transporter activity.
At5g04160	At5g04160	organic anion transmembrane transporter activity.
KMS1	At4g14950	molecular_function_unknown.
PTR2-B	At2g02040	peptide transmembrane transporter activity, peptide:proton symporter activity, oligopeptide transmembrane transporter activity, high-affinity oligopeptide transmembrane transporter activity, tripeptide transmembrane transporter activity, transporter activity.
DIT2.1	At5g64290	oxaloacetate transmembrane transporter activity, L-glutamate transmembrane transporter activity, malate transmembrane transporter activity, transporter activity, oxoglutarate:malate antiporter activity.
UMAMIT18	At1g44800	L-amino acid efflux transmembrane transporter activity, amino acid transmembrane transporter activity, protein binding.
PIP2;3	At2g37180	protein binding, water channel activity.
At3g21690	At3g21690	drug transmembrane transporter activity, antiporter activity, transporter activity.
NPSN13	At3g17440	SNARE binding, SNAP receptor activity.
UTr7	At4g31600	UDP-glucose transmembrane transporter activity, UDP-galactose transmembrane transporter activity.
At3g57280	At3g57280	fatty acid transmembrane transporter activity.
SEP1	At4g34190	chlorophyll binding.
At1g29060	At1g29060	molecular_function_unknown.
VIT1	At2g01770	iron ion transmembrane transporter activity, manganese ion transmembrane transporter activity.
SYP131	At3g03800	SNAP receptor activity, SNARE binding.

PUP11	At1g44750	purine nucleobase transmembrane transporter activity.
NRAMP4	At5g67330	manganese ion transmembrane transporter activity, cadmium ion transmembrane transporter activity, inorganic anion transmembrane transporter activity, metal ion transmembrane transporter activity.
UMAMIT29	At4g01430	L-glutamine transmembrane transporter activity, transmembrane transporter activity.
UTR3	At1g14360	pyrimidine nucleotide-sugar transmembrane transporter activity, UDP-galactose transmembrane transporter activity, UDP-glucose transmembrane transporter activity, antiporter activity.
SYP31	At5g05760	protein binding, SNAP receptor activity, SNARE binding.
VAMP722	At2g33120	SNARE binding, SNAP receptor activity.
PIP2;7	At4g35100	water channel activity, protein binding.
AAP4	At5g63850	amino acid transmembrane transporter activity, acidic amino acid transmembrane transporter activity, neutral amino acid transmembrane transporter activity, primary active transmembrane transporter activity, symporter activity.
SIP1;2	At5g18290	water channel activity.
GLT1	At5g16150	carbohydrate transmembrane transporter activity, glucose transmembrane transporter activity, carbohydrate:proton symporter activity.
At5g23550	At5g23550	molecular_function_unknown.
At1g47530	At1g47530	antiporter activity, transporter activity, drug transmembrane transporter activity.
SYP51	At1g16240	SNAP receptor activity, protein binding, SNARE binding.
Tic20-II	At2g47840	transport.
NIP1;1	At4g19030	arsenite transmembrane transporter activity, water channel activity, protein binding.
AVA-P4	At1g75630	proton-transporting ATPase activity, rotational mechanism, proton transmembrane transporter activity, ATPase activity.
At1g12390	At1g12390	protein binding.
UMAMIT5	At1g75500	transmembrane transporter activity.
PIP1;5	At4g23400	water channel activity, protein binding.
SWEET1	At1g21460	sugar transmembrane transporter activity, protein binding.
UTR1	At2g02810	antiporter activity, UDP-glucose transmembrane transporter activity, UDP-galactose transmembrane transporter activity, pyrimidine nucleotide-sugar transmembrane transporter activity.
TIP3;1	At1g73190	water channel activity.
At1g62880	At1g62880	molecular_function_unknown.
TOM22-V	At5g43970	P-P-bond-hydrolysis-driven protein transmembrane transporter activity.
VAP27	At3g60600	protein binding.
PUP4	At1g30840	purine nucleobase transmembrane transporter activity.
At2g16800	At2g16800	nickel cation transmembrane transporter activity.
ECH	At1g09330	molecular_function_unknown.
PHT4;4	At4g00370	inorganic phosphate transmembrane transporter activity, L-ascorbic acid transmembrane transporter activity, organic anion transmembrane transporter activity, inorganic phosphate transmembrane transporter activity.
CLC-A	At5g40890	protein binding, voltage-gated chloride channel activity, nitrate:proton symporter activity, nitrate transmembrane transporter activity, anion channel activity.
UMAMIT14	At2g39510	L-glutamine transmembrane transporter activity.

ERDL6	At1g75220	glucose transmembrane transporter activity, carbohydrate transmembrane transporter activity, carbohydrate:proton symporter activity.
BET12	At4g14455	SNAP receptor activity, protein transporter activity.
VAMP725	At2g32670	SNAP receptor activity, SNARE binding.
VAMP721	At1g04750	SNAP receptor activity, protein binding, SNARE binding.
SYP43	At3g05710	SNARE binding, SNAP receptor activity.
SYP22	At5g46860	SNAP receptor activity, protein binding, SNARE binding.
VAMP724	At4g15780	SNAP receptor activity, SNARE binding.
YKT61	At5g58060	transport.
SYP21	At5g16830	protein binding, SNAP receptor activity, SNARE binding.
SYP24	At1g32270	SNARE binding, SNAP receptor activity.
SYP32	At3g24350	SNAP receptor activity, SNARE binding.
SYP41	At5g26980	SNAP receptor activity, protein binding, SNARE binding.
SYP61	At1g28490	SNAP receptor activity, protein binding, SNARE binding.
SYP52	At1g79590	SNAP receptor activity, SNARE binding.
SYP112	At2g18260	SNAP receptor activity, SNARE binding.
VTI12	At1g26670	protein binding, SNARE binding, soluble NSF attachment protein activity, SNAP receptor activity.
VAMP713	At5g11150	SNARE binding, SNAP receptor activity.
VTI13	At3g29100	SNARE binding, SNAP receptor activity.
VTI11	At5g39510	protein binding, SNAP receptor activity, SNARE binding, protein binding.
SYP42	At4g02195	protein binding, SNARE binding, SNAP receptor activity.
SNAP33	At5g61210	SNAP receptor activity, protein binding.
SYP124	At1g61290	SNAP receptor activity, SNARE binding.
PHO1-H6	At2g03250	phosphate ion transmembrane transporter activity.
CLC-D	At5g26240	voltage-gated chloride channel activity, protein binding, anion channel activity.
AVT6E	At1g80510	amino acid transmembrane transporter activity.

Supplementary Table 1: Proteins included in the network shown in figure 1.

Primer name	Primer sequence	Template	pDEST plasmid
SECFWATTB1	GGGGACAAGTTTGTACAAAAAAGCAG GCTTTATGAAGACTAATCTTTTTC	GFPgl133Chi, GFPKDEL	pK2GW7
CHIRVATTB2	GGGGACCACTTTGTACAAGAAAGCTGG GTATTACATAGTATCGACTAAAA	GFPgl133Chi	pK2GW7
KDELRVATTB2	GGGGACCACTTTGTACAAGAAAGCTGG GTATCAGAGCTCGTCCTTGGTCG	GFPKDEL	pK2GW7
STFWATTB1	GGGGACAAGTTTGTACAAAAAAGCAG GCTTTATGATTCATACCAACTTGA	Rat Sialyltranferse (first 53 aa)	pK7FWG2
STRVATTB2	GGGGACCACTTTGTACAAGAAAGCTGG GTACATGGCCACTTTCTCCTGGC	Rat Sialyltranferse (first 53 aa)	pK7FWG2
NIP1;1FWATTB1	GGGGACAAGTTTGTACAAAAAAGCAG GCTTAATGGCGGATATCTCGGGA	CDS of AtNIP1;1 (At4g19030)	pK7WGF2, pK7FWG2, pH7WGR2, pBiFCt-2in1 NC, pBiFCt-2in1 NN
NIP1;1RVATTB2	GGGGACCACTTTGTACAAGAAAGCTGG GTATTAAGTGCTACCGATTCTCA	CDS of AtNIP1;1 (At4g19030)	pK7WGF2, pH7WGR2
NIP1;1NOSTOPRV ATTB2	GGGGACCACTTTGTACAAGAAAGCTGG GTAAGTGCTACCGATTCTCACGG	CDS of AtNIP1;1 (At4g19030)	pK7FWG2
NIP1;1RVATTB4	GGGGACAACTTTGTATAGAAAAGTTGG GTTCAAGTGCTACCGATTCTCAC	CDS of AtNIP1;1 (At4g19030)	pBiFCt-2in1 NN
NIP1;1NOSTOPRVATTB4	GGGGACAACTTTGTATAGAAAAGTTGG GTGAGTGCTACCGATTCTCACGGT	CDS of AtNIP1;1 (At4g19030)	pBiFCt-2in1 NC
SYP51FWATTB1	GGGGACAAGTTTGTACAAAAAAGCAG GCTCCATGGCGTCTTCATCGGATT	CDS of AtSYP51 (At1g16240)	pK7WGF2, pH7WGR2
SYP51FWATTB3	GGGGACAACTTTGTATAATAAAGTTGG AATGGCGTCTTCATCGGATTC	CDS of AtSYP51 (At1g16240)	pBiFCt-2in1 NC, pBiFCt-2in1 NN
SYP51RVATTB2	GGGGACCACTTTGTACAAGAAAGCTGG GTATTACATATACTTAACCAACA	CDS and H3 region of AtSYP51 (At1g16240)	pK7WGF2, pH7WGR2, pBiFCt-2in1 NC, pBiFCt-2in1 NN
SYP52FWATTB1	GGGGACAAGTTTGTACAAAAAAGCAG GCTTAATGGCCTCTTCTTCGGATC	CDS of AtSYP52 (At1g79590)	pK7WGF2, pH7WGR2
SYP52FWATTB3	GGGGACAACTTTGTATAATAAAGTTGG AATGGCCTCTTCTTCGGATC	CDS of AtSYP52 (At1g79590)	pBiFCt-2in1 NC, pBiFCt-2in1 NN
SYP52RVATTB2	GGGGACCACTTTGTACAAGAAAGCTGG GTATTACAGGTACTTAACCAGCA	CDS and H3 region of AtSYP52 (At1g79590)	pK7WGF2, pH7WGR2, pBiFCt-2in1 NC, pBiFCt-2in1 NN
SYP61FWATTB3	GGGGACAACTTTGTATAATAAAGTTGC CATGTCTTCAGCTCAAGATCC	CDS of AtSYP61 (At1g28490)	pBiFCt-2in1 NC, pBiFCt-2in1 NN
SYP61RVATTB2	GGGGACCACTTTGTACAAGAAAGCTGG GTATTAGGTCAAGAAGACAAGAA	CDS of AtSYP61 (At1g28490)	pBiFCt-2in1 NC, pBiFCt-2in1 NN
SYP122FWATTB1	GGGGACAAGTTTGTACAAAAAAGCAG GCTTTATGAACGATCTTCTCTCCG	CDS of AtSYP122 (At3g52400)	pK7WGF2, pH7WGR2
SYP122FWATTB3	GGGGACAACTTTGTATAATAAAGTTGC CATGAACGATCTTCTCTCCGGCT	CDS of AtSYP122 (At3g52400)	pBiFCt-2in1 NC, pBiFCt-2in1 NN
SYP122RVATTB2	GGGGACCACTTTGTACAAGAAAGCTGG GTATTAGCGTAGTAGCCGCCGAT	CDS and H3 region of AtSYP122 (At3g52400)	pK7WGF2, pH7WGR2, pBiFCt-2in1 NC, pBiFCt-2in1 NN
51H3FWATTB1	GGGGACAAGTTTGTACAAAAAAGCAG GCTGGATGCGACAAGTTATGAGAG	H3 region of AtSYP51 (At1g16240)	pK7WGF2
51H3FWATTB3	GGGGACAACTTTGTATAATAAAGTTGC CATGCGACAAGTTATGAGAG	H3 region of AtSYP51 (At1g16240)	pBiFCt-2in1 NC, pBiFCt-2in1 NN
52H3FWATTB1	GGGGACAAGTTTGTACAAAAAAGCAG GCTGGATGTTTCAACGGCAAGTTA	H3 region of AtSYP52 (At1g79590)	pK7WGF2
52H3FWATTB3	GGGGACAACTTTGTATAATAAAGTTGC CATGTTTCAACGGCAAGTTA	H3 region of AtSYP52 (At1g79590)	pBiFCt-2in1 NC, pBiFCt-2in1 NN
122H3FWATTB3	GGGGACAACTTTGTATAATAAAGTTGC CATGTCGGACCGGCAAAGAA	H3 region of AtSYP122 (At3g52400)	pBiFCt-2in1 NC, pBiFCt-2in1 NN

Supplementary Table 2: List of primers used.

Supplementary Figures

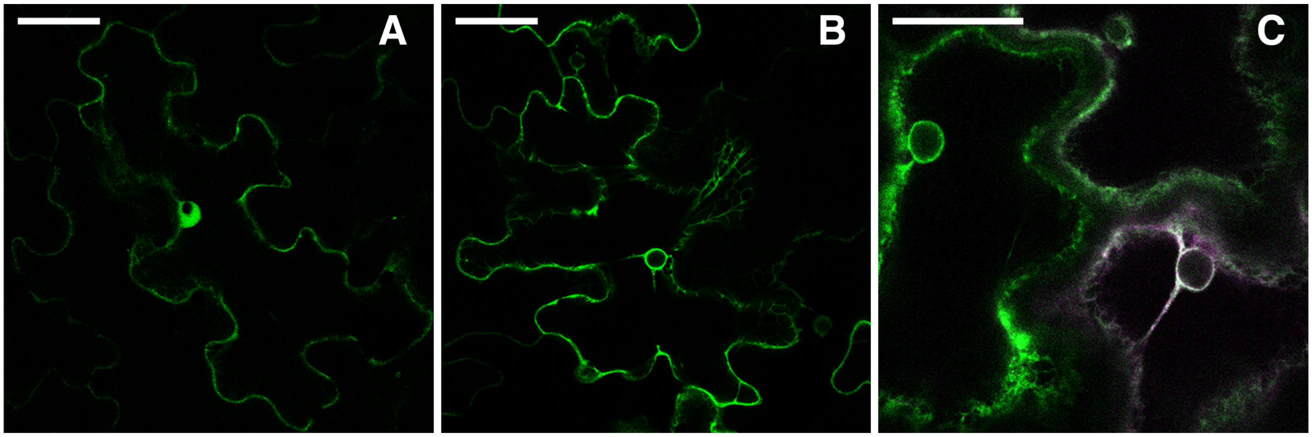

Supplementary Figure 1: Transient expression in Nicotiana tabacum epidermis of A) NIP1;1::GFP with cytosolic distribution; B) GFP::NIP1;1 associated to the ER; C) GFP::NIP1;1 co-expressed with RFP::NIP1;1. Transformation was performed as previously described (Di Sansebastiano et al. 2004). Scale bar = 20 µm.

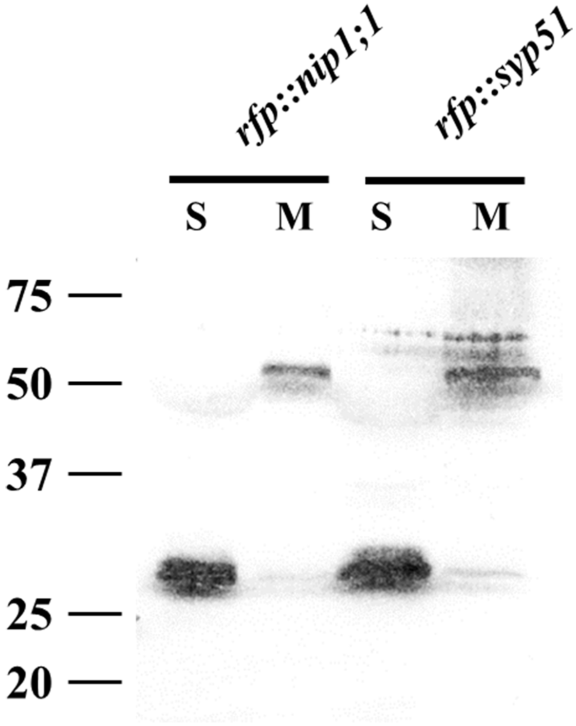

Supplementary Figure 2:
Proteins were analyzed (Renna et al., 2013) by immunodetection on Western-blot using RFP antibodies (ab62341 Abcam) to confirm RFP::SYP51 and RFP:NIP1;1 overexpression. S= soluble fraction, M= membrane fraction. The numbers in the right represent the relative molecular weight in KDa.

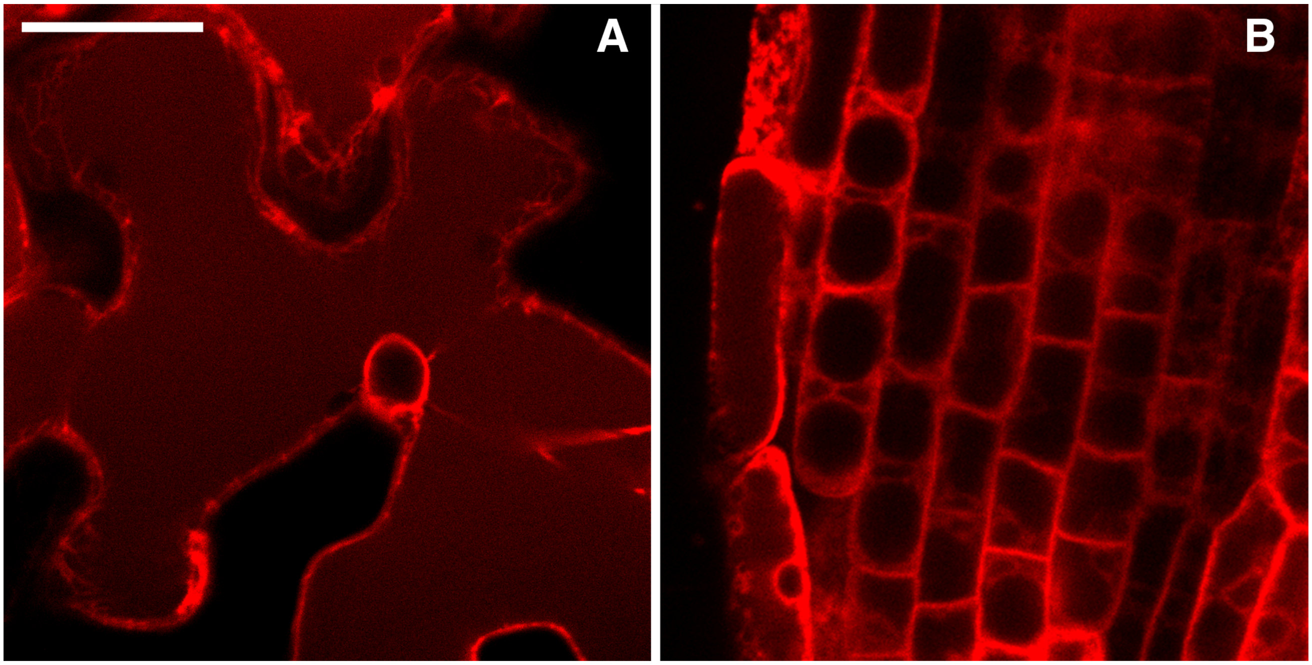

Supplementary Figure 3: Confocal images of cells in a transgenic Arabidopsis plant expressing RFP::NIP1;1**, A)** in the leaf epidermis; **B)** in root cells. Scale bar = 20 µm

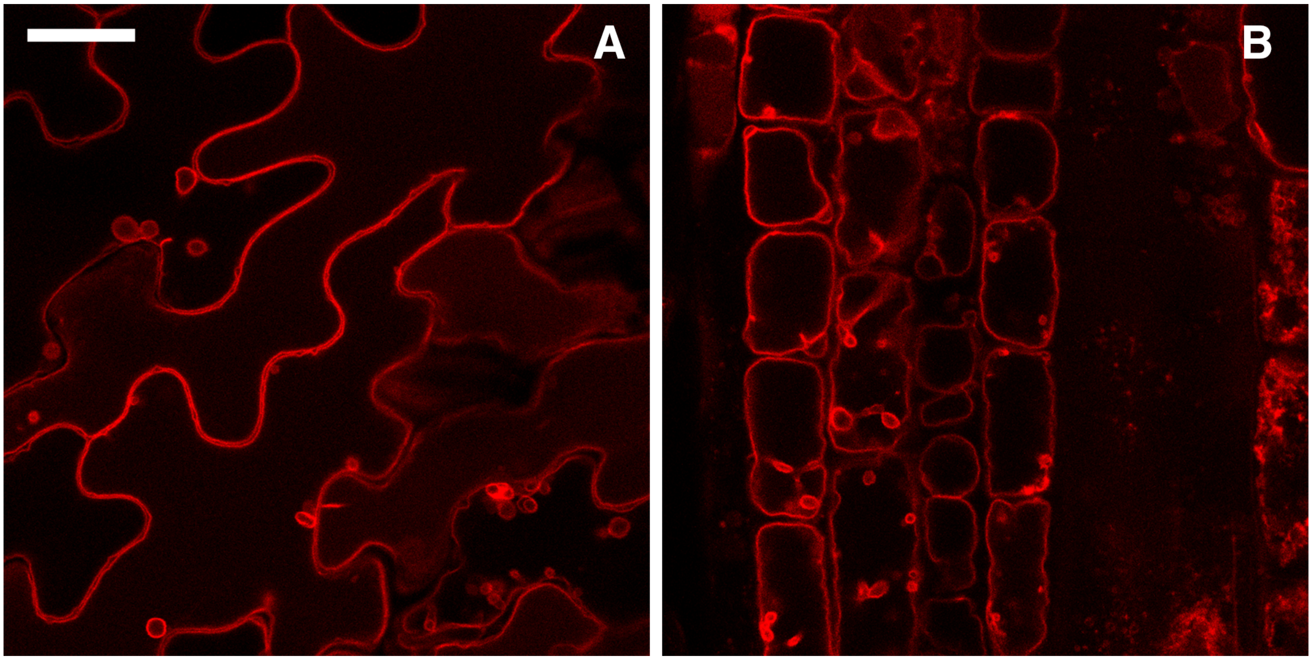

Supplementary Figure 4: Confocal images of cells in a transgenic Arabidopsis plant expressing RFP::SYP51**, A)** in the leaf epidermis; **B)** in root cells. Scale bar = 20 µm

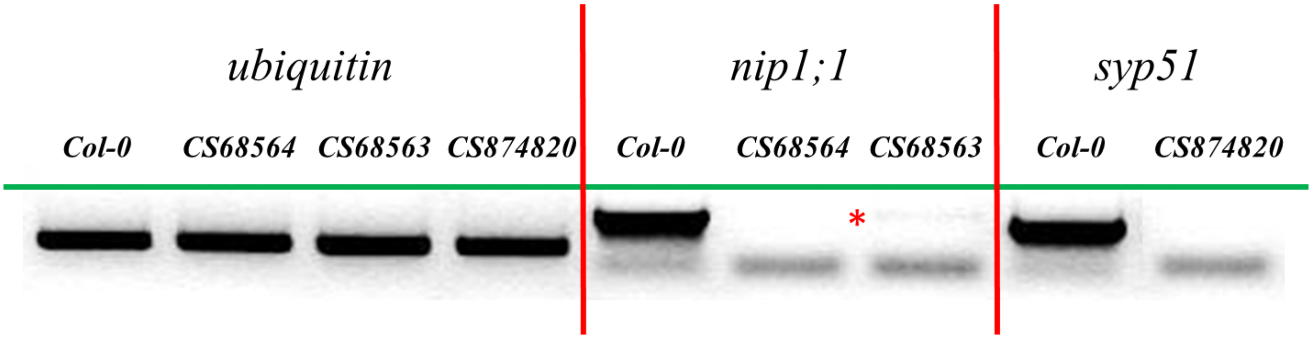

Supplementary Figure 5: To confirm the expression suppression of genes in transgenic plants, RNA was isolated from 2-week- old seedlings (RNeasy Plant Mini Kit; Qiagen). The first-strand cDNA was then synthesized using the SuperScript III first-strand synthesis system (Invitrogen). PCR was performed using the High- fidelity DNA Polymerase (Finnzymes, Espoo, Finland) for 35 cycles. For an internal control, the constitutive gene Ubiquitin10 was used. * evidences highly reduced but not suppressed expression of nip1;1.

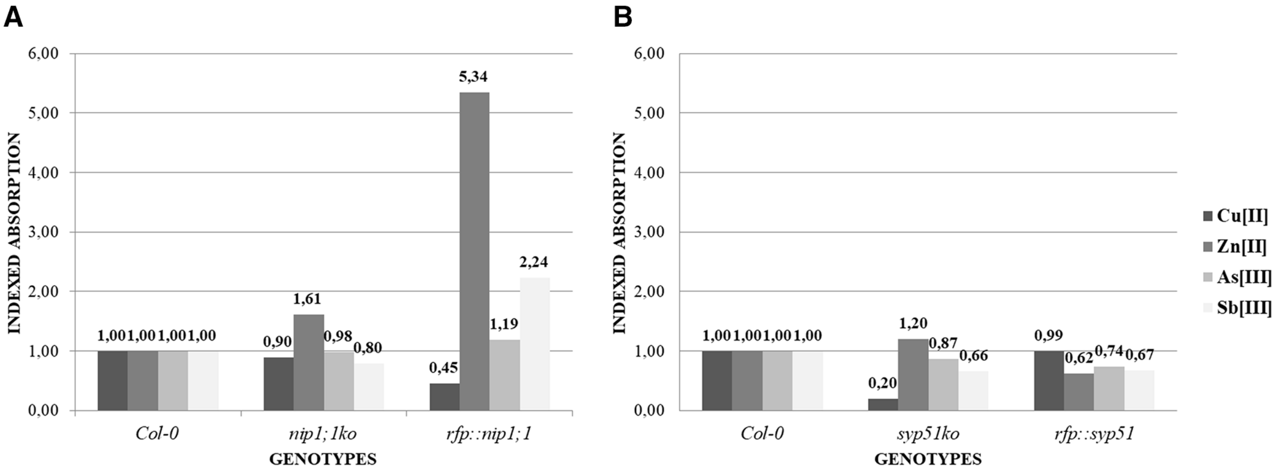

Supplementary Figure 6: Indexed Cu[II], Zn[II], As[III] and Sb[III] uptake by control and trasngenic plants.

Supplementary video 1: Tonoplast “bubbling” in Arabidopsis epidermal cell during co-expression of GFP::SYP51 (green) and RFP::NIP1;1 (red). Yellow signal evidence co-localization of fluoreochromes in aggregates at the bubbling site.
